# Clonal heterogeneity in ER+ breast cancer reveals the proteasome and PKC as potential therapeutic targets

**DOI:** 10.1038/s41523-023-00604-4

**Published:** 2023-12-02

**Authors:** Lukas Beumers, Efstathios-Iason Vlachavas, Simone Borgoni, Luisa Schwarzmüller, Luca Penso-Dolfin, Birgitta E. Michels, Emre Sofyali, Sara Burmester, Daniela Heiss, Heike Wilhelm, Yosef Yarden, Dominic Helm, Rainer Will, Angela Goncalves, Stefan Wiemann

**Affiliations:** 1 Division of Molecular Genome Analysis, German Cancer Research Center (DKFZ), Im Neuenheimer Feld 580, 69120 Heidelberg, Germany; 2 Faculty of Biosciences, University of Heidelberg, Im Neuenheimer Feld 234, 69120 Heidelberg, Germany; 3 Division of Somatic Evolution and Early Detection, German Cancer Research Center (DKFZ), Im Neuenheimer Feld 580, 69120 Heidelberg, Germany; 4 Department of Immunology and Regenerative Biology, Weizmann Institute of Science, Rehovot, 76100 Israel; 5 Proteomics Core Facility, German Cancer Research Center (DKFZ), Im Neuenheimer Feld 580, 69120 Heidelberg, Germany; 6 Cellular Tools Core Facility, German Cancer Research Center (DKFZ), Im Neuenheimer Feld 580, 69120 Heidelberg, Germany

**Keywords:** Experimental models of disease, Cancer models

## Abstract

Intratumoral heterogeneity impacts the success or failure of anti-cancer therapies. Here, we investigated the evolution and mechanistic heterogeneity in clonal populations of cell models for estrogen receptor positive breast cancer. To this end, we established barcoded models of luminal breast cancer and rendered them resistant to commonly applied first line endocrine therapies. By isolating single clones from the resistant cell pools and characterizing replicates of individual clones we observed inter- (between cell lines) and intra-tumor (between different clones from the same cell line) heterogeneity. Molecular characterization at RNA and phospho-proteomic levels revealed private clonal activation of the unfolded protein response and respective sensitivity to inhibition of the proteasome, and potentially shared sensitivities for repression of protein kinase C. Our in vitro findings are consistent with tumor-heterogeneity that is observed in breast cancer patients thus highlighting the need to uncover heterogeneity at an individual patient level and to adjust therapies accordingly.

## Introduction

Breast cancer remains the most commonly diagnosed malignancy in women worldwide^1^ and comprises a heterogeneous group of diseases characterized by distinct clinical, histopathological, and molecular features^2^. Accordingly, breast tumors are initially classified by immunohistochemistry (IHC) based on the expression of estrogen receptor (ER), progesterone receptor (PR), human epithelial growth factor receptor 2 (HER2) and the proliferative marker Ki-67^3^. Around 60–70% of tumors are characterized by ER expression^4^. Patients with ER+ tumors are efficiently treated with endocrine therapies to abrogate the tumor promoting effects of estrogen^5^, leading to overall survival rates of over 90%^6^. First-line endocrine therapy consists of selective ER modulators (SERMs) such as Tamoxifen and of aromatase inhibitors (AIs) for pre- and postmenopausal patients, respectively. Despite high therapeutic efficacy, up to 40% of patients with late-stage diagnoses relapse within 20 years of treatment start^7^ and recurrences can be caused by diverse resistance mechanisms^8^. Along these lines, we recently identified ATF3, a member of the ATF/CREB family of transcription factors, as novel driver of endocrine therapy resistance in vitro and in vivo^9^, and pinpointed candidate methylation sites which are clinically associated with endocrine therapy resistance^10^.

Tumor cell heterogeneity contributes to therapy resistance by increasing the likelihood of cells having a resistant phenotype or the capacity to acquire such a resistant phenotype are present prior to treatment^11,12^. Next-generation sequencing (NGS) has fundamentally uncovered genetic heterogeneity also in breast tumors^13,14^. However, tumor heterogeneity and response to therapy can be driven by genetic, epigenetic and stochastic events in the tumor cells, and by local or systemic factors such as tissue context and clonal expansion in healthy tissue^15,16^. Stochasticity in gene expression and other mechanisms triggering protein abundance may lead to variable protein expression states among individual cells and induce heterogeneity of cellular phenotypes^17^. Tumor heterogeneity may destine therapeutic resistance via two mechanisms: Pre-existent resistant clones may be selected through the applied therapy (i.e., primary resistance)^18,19^ or initially treatment-persisting cells may adapt to and then acquire resistance to the applied therapy (secondary resistance)^20^. While the former mechanism points at the presence of alterations that confer a resistance phenotype in some tumor cells before therapy, the latter is rather connected to stochastic events that are selected and then fixed in tumor cells carrying eventually dominating molecular states.

Here, we leveraged cellular barcoding, cell cloning and integrative multi-omics analysis to disentangle clonal adaptions to endocrine therapies. We barcoded two treatment-sensitive ER+ breast cancer cell lines and then rendered them resistant to Tamoxifen (TAMR) or long-term estrogen deprivation (LTED), the latter mimicking clinically applied aromatase inhibition. We find evidence of both, the selection of pre-existing resistant clones and for acquired resistance in persister cell populations. Individual clonal populations were isolated and characterized at the phenotypic, gene expression (RNA-Seq) and phospho-proteome levels. There, we identified private and potentially shared contributors to resistance, opening potential therapeutic vulnerabilities. Sensitivity to the proteasome inhibitor Bortezomib was private and correlated with activation of the unfolded protein response (UPR). In contrast, protein kinase C (PKC) isoforms were commonly activated in TAMR and LTED clones originating from one cell line, while treatment with the pan PKC inhibitor Sotrastaurin was significant just with one LTED clone. Clinical relevance of our findings was established in patient-oriented analysis of the CPTAC-BRCA proteogenomic cohort^21^ upon stratification by ER− vs ER+. PKC activation in particular, was evident in two patients with ER− disease, which closely resembled our in vitro models. Taken together, we developed cellular models of endocrine resistance that revealed private and potentially shared contributors to endocrine therapy resistance thus supporting the need for molecular stratification at the single patient resolution for treatment of advanced luminal breast cancer.

## Results

### Clonal in vitro models of endocrine therapy resistance

In this study, we wanted to disentangle the heterogeneity of endocrine therapy resistance in individual clones using in vitro models. Molecular changes should be uncovered at the gene expression and phospho-proteomic levels, and new potential vulnerabilities be identified. To this end, we transduced treatment-naïve ER+ breast cancer cell lines MCF7 and T47D with the ClonTracer library^22^ at an MOI of 0.05 to favor single barcode integration. Following short-time expansion, cultures were split into twenty replicates for every cell line: Five replicates of each were frozen down to later assess the barcode complexity in the initial cell population (Supplementary Fig. 1). Five replicates of each cell line were treated for eight months with either 4-OHT or estrogen deprivation (-E2) to induce Tamoxifen resistant (TAMR) and long-term estrogen deprived (LTED) cell lines, respectively. The latter mimics aromatase inhibition. Control cells were kept in standard media (+E2). Tamoxifen reduced the proliferation of cells, however, did not induce a quiescent state. In contrast, cells kept in estrogen deprived conditions entered a quiescent state for several months and re-gained proliferative capacity after six months.

**Fig. 1:**
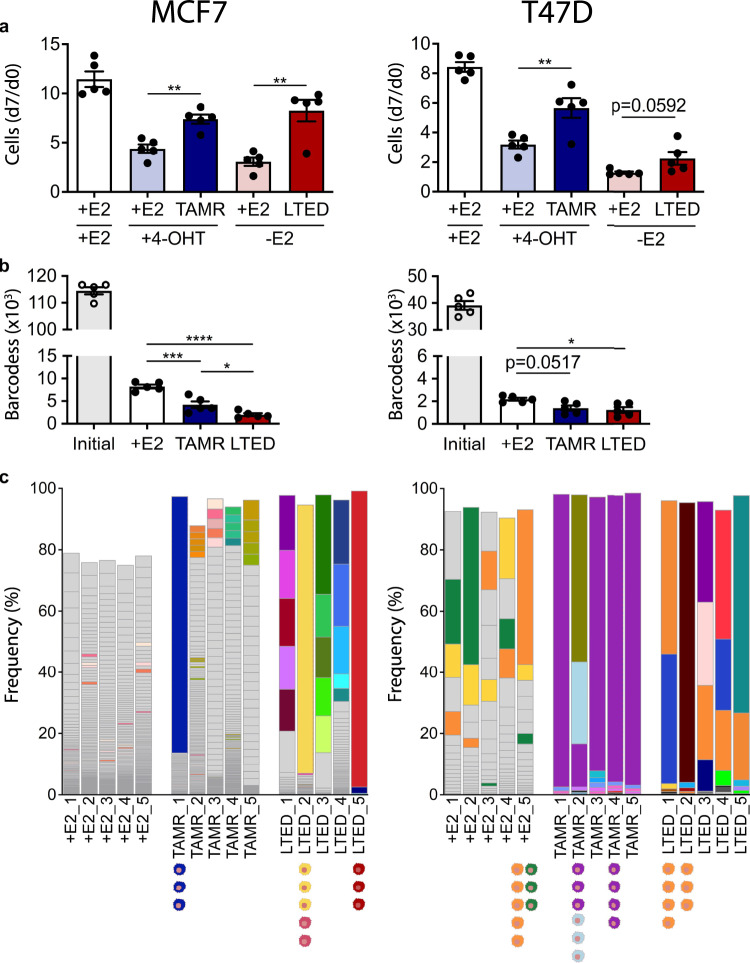
Initial analysis of barcoded endocrine resistance models. MCF7 and T47D cell lines were barcoded and then resistance was induced over eight months to either 4 hydroxy-tamoxifen (4-OHT) or estrogen deprivation (-E2), in five replicates each, to render them tamoxifen resistant (TAMR) or long term estrogen deprived (LTED). Barcoded control (+E2) replicates were cultivated in media with estrogen for the same period. **a** After eight months of culture in respective media, proliferation was measured in all replicates of MCF7 (left) and T47D (right) by microscopy-based nuclei counting at day 0 (d0) and day 7 (d7) with cells grown in indicated conditions (+E2, 4-OHT, -E2). Additionally, +E2 barcoded cells were pre-treated for 14 days without estrogen. The average (mean) ± SEM of five individual barcoded replicates (*n* = 1 with 6 technical replicates each) per condition is shown. Significance levels: *p < 0.05, **p < 0.01, ***p < 0.001 as determined by unpaired two-tailed *t*-test. **b** Barcodes were sequenced in each of the initial, +E2, TAMR, and LTED replicates. Indicated are mean ± SEM for respective conditions. *p < 0.05, ***p < 0.001, ****p < 0.0001 as determined by one-way ANOVA with Dunnett multiple comparisons test. **c** The five and 25 most abundant barcodes in each replicate for the +E2, TAMR, and LTED conditions of MCF7 and T47D cell lines, respectively, are indicated in different colors and are also indicated in the +E2 conditions if enriched. Other barcodes are shown in gray. Individual clones were isolated from selected replicates and are indicated with color-coding to represent the respective barcodes.

At eight months of cultivation, treated replicates had regained a more proliferative phenotype compared to +E2 replicates that had been challenged with endocrine therapies only for a short time, indicating successful induction of endocrine resistance in the former (Fig. 1a). Bulk sequencing of barcodes in every replicate discerned the barcode- and the inferred clonal complexities. At least 5 × 10^6^ barcode reads were obtained for all cell lines (Supplementary Table 1). The theoretical maximum of approximately 1*10^5^ transduced barcodes was found in the initial cell population of the barcoded MCF7 cell line (1.15*10^5^ unique barcodes), while the complexity was lower (4*10^4^ unique barcodes) in T47D (Fig. 1b). Prolonged cultivation of replicates led to a strong reduction in the complexity of barcodes even in the absence of endocrine therapy (i.e., the +E2 controls). Treatment with 4-OHT and particularly with estrogen deprivation (-E2) induced a significant further decrease in the numbers of distinct barcodes, however, also an enrichment of a few barcodes, while no barcodes were enriched in the initial barcode library (Supplementary Fig. 2, Supplementary Files 1–3). This supports the intended rather homogenous representation of barcodes in the initial barcoded pool as well as neutral drift potentially having occurred in some replicates.

Next, we inspected the identity of barcodes in the respective replicates and found extensive heterogeneity regarding numbers and recurrence of barcodes between individual replicates. In Fig. 1c, for MCF7 the five and for T47D the 25 most abundant barcodes in each replicate are color coded. Other barcodes are shown in gray. The apparent barcode- and clonal complexity appeared to be higher in MCF7 than in T47D. In the MCF7 TAMR replicates only 16 barcodes along all TAMR replicates were enriched with a frequency greater than five percent. None of those barcodes was enriched in more than one replicate, pointing to resistance acquisition of distinct clones that were selected during treatment. In contrast, 4/5 replicates in the T47D TAMR model were dominated by a clone carrying the *purple* barcode and this was prominent also in the fifth replicate (Fig. 1c). The recurrent enrichment of clones carrying the *purple* barcode could suggest that the respective cells had been Tamoxifen-resistant already prior to the onset of treatment as this barcode was overrepresented neither in the T47D + E2 controls nor in the LTED conditions (Supplementary Fig. 2).

We then isolated single clones from selected replicates (Supplementary Fig. 3a and Fig. 1c). The barcodes in these clones were sequenced and associated with the same color code that was used for the whole replicate. At least three clones carrying the identical barcodes could be isolated from two independent replicates each of the T47D TAMR and LTED conditions. Clones carrying the *orange* barcode were isolated from two LTED replicates a well as from the +E2 control replicate. While clonal populations having been isolated from the same replicate had been cultivated independent of one another for a period of at least six months, clones isolated from different replicates of any condition had been cultivated independently for at least 14 months (compare Supplementary Fig. 1).

Barcode sequencing of individual clones revealed that most T47D clones carried only one barcode, as was expected given the MOI of 0.05 that had been used in the generation of the barcoded libraries. However, the *purple* T47D TAMR clones carried two barcodes that were found at the same ratio in all replicates. The MCF7 *blue* TAMR_1 clones, for example, had even 17 barcodes integrated into their genomes (Supplementary Fig. 3b), requiring an NGS approach to identify all barcodes. A list of clones and their integrated barcodes is provided in Supplementary Table 2. Some barcodes detected in sequencing of MCF7 clones were not detected in the initial barcoded cell pools, potentially because some barcodes did not match the expected pattern of ([AT][GC])x15^22^. As we determined the barcodes in individual clones only for those MCF7 replicates and clones that are indicated in Fig. 1c, the apparent clonal complexity in other replicates is likely exaggerated in the figure. Yet, we refrained from isolating single clones from the MCF7 control (+E2) and instead, employed pools of three MCF7 + E2 replicates (i.e., +E2_1, +E2_3, and +E2_5) as controls in any experiments using MCF7 cells throughout the study.

Finally, we wanted to uncover the chromosome positions virus-encoded barcodes had integrated into, to potentially exclude viral integration into genomic loci as drivers of resistance. To this end, we amplified and sequenced integration sites in MCF7 replicates TAMR_2 and LTED_5 as well as T47D replicates +E2_2, TAMR_2, and LTED_2. Read counts of LAM-PCR^23^ libraries (File ‘Integration site analysis.xlsx’ at Zenodo)) were matched with barcode read counts in the same replicates suggesting viral integration sites for particular barcodes and clones. Matches between integration sites and barcodes were validated for the individualized clones using PCR (Supplementary Fig. 3c–f). None of the viral integration sites (Supplementary Table 3) were associated with significant expression changes of nearby genes (|log2FC| ≥ 0.05 and p-adj. ≤ 0.05), except for integration of a barcode into Chr11:31,426,888 (GRCh38/hg38) in the *orange* barcode in T47D LTED and +E2 clones. This barcode had integrated into intron 4 of *DNAJC24*. The gene was significantly higher expressed in T47D *orange* LTED clones compared to *orange* + E2_5 clones (data not shown). Since the *orange* clonal populations were all characterized by the same barcode and integration site, however, upregulation of *DNAJC24* expression was likely a treatment-specific event (LTED vs +E2) and not related to the viral integration. We, therefore, excluded viral integration and potential deregulation of genes in close proximity to the viral integration sites as potential drivers of endocrine therapy resistance for the isolated clones.

### Gene expression are stable over time in endocrine resistant clones

**Fig. 2:**
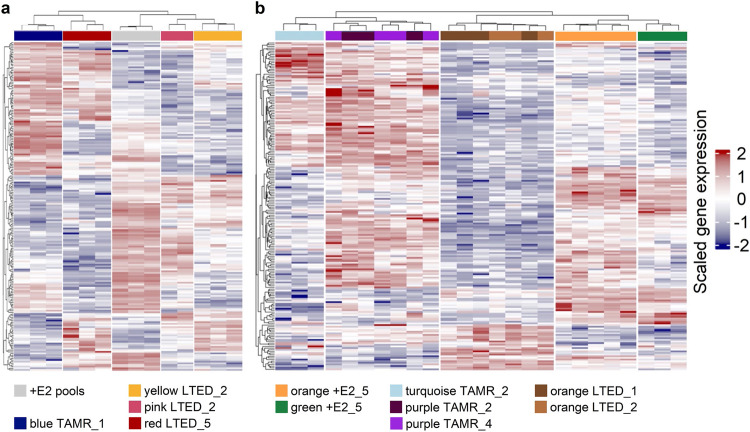
Gene expression trajectories are stable in clonal lineages. RNA-sequencing was performed with 2 to 5 clones sharing the same barcodes from the indicated replicates. Barcodes are color-coded. Heatmaps of the top 200 most variable genes were generated using the R package ComplexHeatmap (version 2.10.0)^71^ for **a** MCF7 and **b** T47D cell lines. Hierarchical clustering was applied in both genes (rows) and cell lines (columns) (Euclidean distance, complete linkage). The illustrated values are scaled log2-counts per million (CPM) TMM normalized values.

Next, we wanted to know if the observed heterogeneity of clones in the different conditions and replicates was mirrored at the gene expression level. To this end, RNA was sequenced from clones sharing the same as well as having different barcodes. Unsupervised hierarchical clustering of the top 200 most variable genes across all samples in each dataset (Supplementary Files 4 and 5) revealed stable clonal lineages, however, also extensive heterogeneity between clonal populations having different barcodes. Individual clones sharing the same barcode could be robustly discriminated from clones that had any other barcode, both for MCF7 (Fig. 2a) and T47D (Fig. 2b). This suggests that gene expression patterns were stable in clones sharing the same barcode even though the span between clone isolation and molecular profiling was at least six months of continuous culture. While this might be expected for clones isolated from the same replicate, this was also apparent for clones from different replicates thus having been cultured independently for at least 14 months (compare Supplementary Fig. 1). This could implicate that the respective constant stressor (i.e., 4-OHT and estrogen-deprivation, respectively) induced fixation of particular, yet for different clones individual, gene expression patterns.

### Clones having derived from the same parental cell display distinct phenotypes after selection

**Fig. 3:**
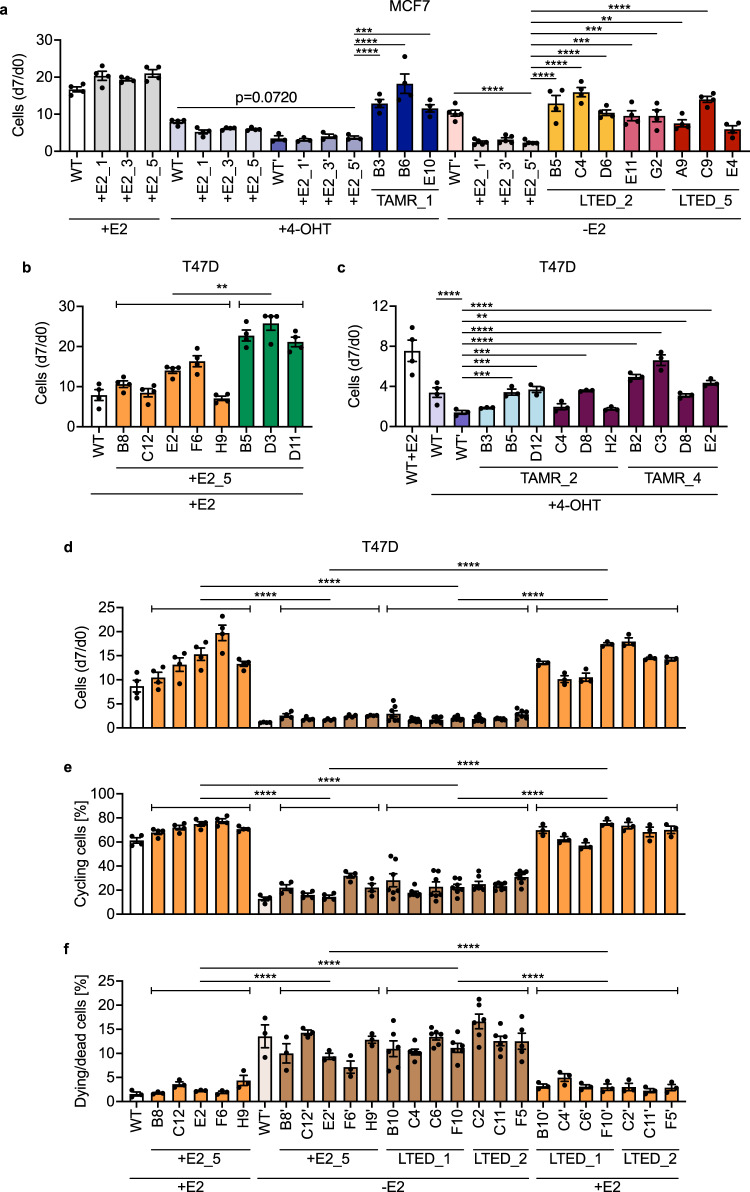
MCF7 and T47D TAMR and LTED clones display distinct phenotypes. **a** Proliferation of wildtype MCF7 cells (WT), three control +E2 replicates, and isolated clones representing *blue* TAMR_1, *yellow* and *pink* LTED_2, and *red* LTED_5 clones was measured by microscopy-based nuclei counting at day 0 (d0) and day 7 (d7) of culture with indicated treatments (+E2, 4-OHT, -E2). Barcoded control (+E2) replicates were cultivated without treatment for the same period of time. ‘indicates 14 day pre-treatment with 4-OHT or by estrogen deprivation (-E2) and then continued treatment for the duration (7 days) of the proliferation assay. **b** Proliferation of wildtype T47D cells (WT) and *orange* as well as *green* clones isolated from the T47D + E2_5 replicate, grown in media with estrogen (+E2). **c** Proliferation of indicated clones in media with 4OH-tamoxifen (+4-OHT). **d** Proliferation of T47D *orange* clones isolated from +E2_5, LTED_1, and LTED_2 replicates, grown in media containing estrogen (-E2) or deprived of estrogen (-E2). (**e**, **f**) Percentages of cycling (**e**) and dying/dead cells (**f**) after 4 days of indicated treatment (+E2, −E2). ‘indicates 14 day pre-treatment with estrogen (+E2), 4-OHT, or estrogen deprivation (−E2) and then continued treatment for the duration (4 or 7 days) of the assays. *n* ≥ 3 with ≥5 technical replicates each for all assays. Shown are means ± SEM. Clones with the same barcode originating from the same treatment were grouped and the grouped clones compared. *p < 0.05, **p < 0.01, ***p < 0.001 and ****p < 0.0001 as determined by unpaired two-tailed *t*-test (**b**) or by one-way ANOVA with Dunnett multiple comparisons test (**a**, **c**–**f**).

Having identified stable clonal trajectories in the RNA-Seq data, we next assessed the proliferative capacity of individual clones. Here, we observed similar but distinct proliferative capacities of clones even when these carried the same barcodes and had clustered in the RNA-seq experiment (Fig. 3a–c). Hence, the rather homogenous gene expression patterns present in clones carrying the same barcodes were not mirrored by a similar homogenous distribution of proliferative phenotypes. Next, we focused specifically on the *orange* clones, where we had isolated five clones from the +E2_5, and four and another three clones from the LTED_1 and LTED_2 replicates of the T47D cell line, respectively. These clones had all derived from the same initial cell as they carried the same barcode integrated into the identical chromosomal location (Fig. 3f). We tested the clones in cellular assays informing on proliferation, cell cycle progression and apoptosis (Fig. 3d–f). This revealed consistent clonal patterns within and also between replicates of the same condition (i.e., LTED_1 and LTED_2). The *orange* barcoded T47D + E2_5 showed robust proliferation, however, this was strongly reduced upon short-term estrogen deprivation. The respective T47D LTED clones showed a similar phenotype having limited proliferative capacity when grown without estrogen. Conversely, strong proliferation could be induced by short-term supplementation of estrogen (Fig. 3d). The short- and long-term estrogen deprived *orange* barcoded T47D clones thus displayed a ‘cycling persister’ phenotype^24^ characterized by a reduced but prominent fraction of cycling cells and a strong increase in dying/dead cells compared to non-treated counterparts from the same clonal origin (Fig. 3e, f). Given the rather similar growth patterns observed with *orange* + E2 and LTED clones, we next utilized our RNA-Seq data to identify deregulated pathways.

### Pathway activities in MCF7 and T47D TAMR and LTED clones reflect intra- and inter-tumor heterogeneity

**Fig. 4:**
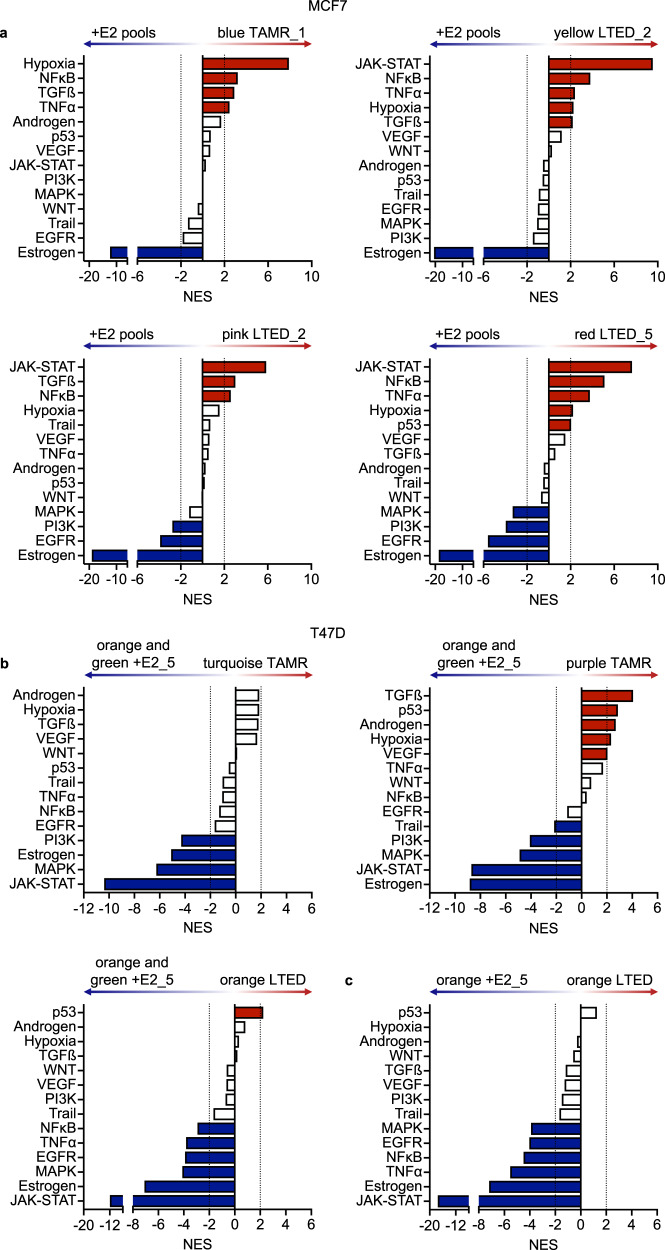
Pathway analysis of clonal populations reveals cell line- (MCF7 vs. T47D), treatment- (TAMR vs. LTED), and clone-specific activities. RNA-Seq data from MCF7 (**a**) and T47D (**b**, **c**) + E2 controls (data from pool of three replicates for MCF7, combined data from *orange* and *green* clones (b) or orange clones (c) from respective replicates +E2_5 for T47D) was analyzed to estimate pathway activities for indicated clones using PROGENy^25,26^. Normalized enrichment scores (NES) of differentially regulated pathways with significantly higher (red) and lower (blue) predicted relative activities in the respective resistance conditions compared to the indicated +E2 controls are presented. A NES of +/− 2 was taken as cut-off for significance. Clones having been isolated from the same replicates and characterized by the same barcodes were treated as biological replicates.

Here, wanted to investigate if the differences in clonal gene expression patterns were reflected by similar differences in pathway activities and if these could hint at common or private molecular contributors to drug resistance. To this end, we considered clones isolated from the same replicate and sharing the same barcode as biological replicates. PROGENy^25,26^ was employed with RNA-seq data to estimate the activity of 14 major signaling pathways in drug-treated cells compared to untreated controls. Estrogen signaling was significantly repressed in all clones (Fig. 4), reflecting the therapeutic block imposed by 4-OHT and estrogen deprivation. Mutation of *ESR1* has been determined as one mechanism of acquired endocrine therapy resistance^27^. Consistent with the observed downregulation of ER−signaling in our resistant models, we did not observe *ESR1* hotspot mutations in any endocrine therapy resistant clones (Supplementary Fig. 4). Hence, we next focused on other pathways and mechanisms that might be common or contribute to resistance just in particular clones.

JAK-STAT signaling was strongly upregulated only in the LTED clones of MCF7 (Fig. 4a), while this pathway was consistently downregulated in all T47D clones (Fig. 4b). Hence, this regulation was different for the two treatments in MCF7, and in opposite directions for the two cell lines. Similarly, a significant activation of NFκB signaling was shared among all MCF7 TAMR and LTED conditions, while this pathway was not changed or even downregulated in T47D TAMR and LTED clones. Both, JAK-STAT and NFκB signaling have been associated with the tumor (immune) microenvironment^28,29^.

In summary, we have uncovered substantial commonalities in pathway activities between cell lines and treatment conditions, however, also specific differences that might be related to inter- and intra-tumor heterogeneity. We next wanted to test potential common as well as private therapeutic vulnerabilities in the endocrine therapy-resistant clones, focusing on the MCF7 TAMR and LTED cell models.

### Sensitivity to the proteasome inhibitor Bortezomib correlates with unfolded protein response as a private vulnerability

To find therapeutic vulnerabilities, we next estimated transcription factor activities using DoRothEA^30^ and decoupleR^31^. Furthermore, we analyzed kinase activities using matched phosphoproteomic data with Omnipath^32^ and decoupleR^31^. The latter resulted in the identification of at least 17,000 phosphosites (class 1, localization probability >95%) in every sample (MCF7: 20,207–25,874; T47D: 17,118–24,449). Of these, between 46–74% were shared between all clones sharing identical barcodes (Supplementary Fig. 5a). Additionally, unsupervised hierarchical clustering of all consistently identified phosphopeptides showed that clones with the same barcode clustered together (Supplementary Fig. 5b), corroborating also our results from gene expression analysis (see Fig. 2).

We had found NFκB signaling differentially regulated in endocrine therapy resistant MCF7 vs. T47D clones (compare Fig. 4a, b). Analysis of phospho-proteomic data however did not reveal patterns indicating potential activation of the IκB kinases IKKα, IKKβ, IKKε or TBK1 (Fig. S6a) suggesting that potential pathway activation should be downstream of IκB kinases. The key downstream transcription factor p65/*RELA*^33^ was estimated from the RNA-Seq data to be relatively activated in MCF7 LTED clones and, to a lesser extent in the TAMR clone (Fig. S6b). Knock-down of *RELA* (Fig. S6c) induced similar phenotypes in the endocrine sensitive +E2_5 cells and in TAMR as well as LTED clones as proliferation and cell viability as well as the percentage of cycling cells were consistently decreased while dying/dead cells were increased (Fig. S6d). These findings point at an essential role for p65 even in endocrine sensitive cells, suggesting that complete inhibition of NFκB signaling might be too harsh in view of a potential window of therapeutic targeting.

**Fig. 5:**
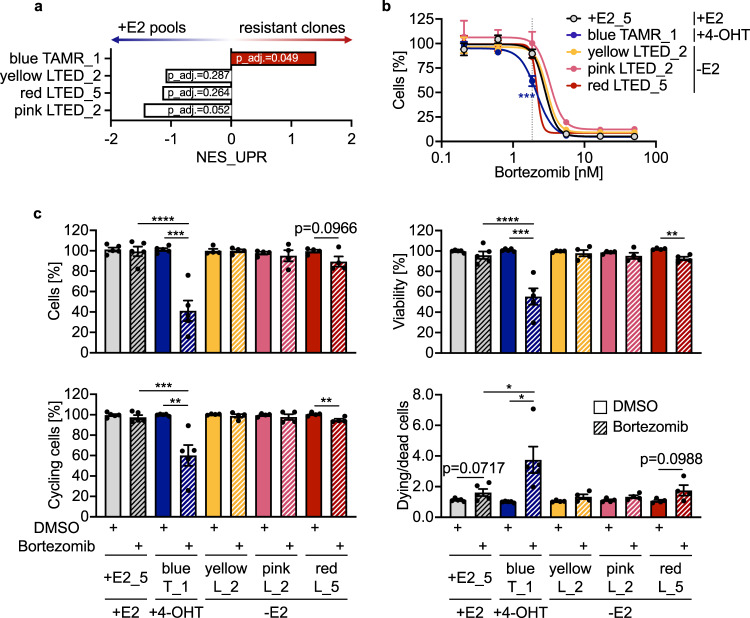
Sensitivity to Bortezomib treatment correlates with activation of the unfolded protein response (UPR) in MCF7 models. **a** Relative activation of the UPR was tested applying GSEA^57^ with RNA-seq data to MCF7 TAMR and LTED clones compared to the +E2 control pools. **b** Indicated clones were kept in the specified media (+E2, +4-OHT, −E2) and treated with increasing concentrations of Bortezomib. Cell numbers were determined by microscopy-based nuclei counting at day 0 (d0) and day 7 (d7) of culture and normalized to the DMSO controls (*n* = 3 with 3 technical replicates each). Gray dotted line indicates effect of 1.85 nM Bortezomib. *p < 0.05, **p < 0.01 as determined by two-way ANOVA with Dunnett multiple comparisons test. **c** Same clones (same color coding as in **b**) were treated with DMSO (solid filling) or 1.85 nM Bortezomib (shaded filling). Nuclear cell count and ATP levels were determined 6 days after treatment start while EdU and DAPI incorporation were quantified 4 days after treatment start. Results were normalized to DMSO controls. *n* ≥ 4 with ≥4 technical replicates. Shown are mean ± SEM. *p < 0.05, **p < 0.01, ***p < 0.001, ****p < 0.0001 as determined by unpaired two-tailed *t*-tests (DMSO vs. Bortezomib treatment) or one-way ANOVA with Dunnett multiple comparisons test (Bortezomib treated conditions).

In contrast, MCF7 *blue* TAMR_1 clones also showed a significant enrichment in the expression of genes that are connected to the unfolded protein response (UPR), pointing to its activation (Fig. 5a) and to a potential dependency on the proteasome. Accordingly, we utilized the proteasome inhibitor Bortezomib which is applied to treat NFκB-driven multiple myeloma^34^ and is hypothesized to abrogate also the UPR^35^. We initially assessed the sensitivity for Bortezomib in MCF7 clones and one +E2 replicate (+E2_5), which revealed the highest sensitivity (Fig. 5b) to Bortezomib treatment for the MCF7 *blue* TAMR_1 cells that had also shown a predicted activation of UPR. No association between activation of NFκB signaling and sensitivity to Bortezomib was evident. We next assessed the effects Bortezomib treatment on cell proliferation, viability, and the ratios of cycling cells in the MCF7 TAMR and LTED clones (Fig. 5c). We used Bortezomib at a 1.85 nM concentration as we had observed the strongest difference in sensitivities for the respective cell clones there. A strong and significant effect was only detected in the *blue* TAMR cells while the other cells were not at all or just slightly (*red* LTED_5) affected. Taken together, sensitivity to Bortezomib correlated with activation of the UPR just in the MCF7 *blue* TAMR_1 clones thus highlighting activation of the proteasome and inhibition by Bortezomib as private and druggable endocrine therapy resistance contributor to a more complex, multifactorial resistance phenotype.

### PKC activation as potentially shared contributor to endocrine therapy resistance in different clones

**Fig. 6:**
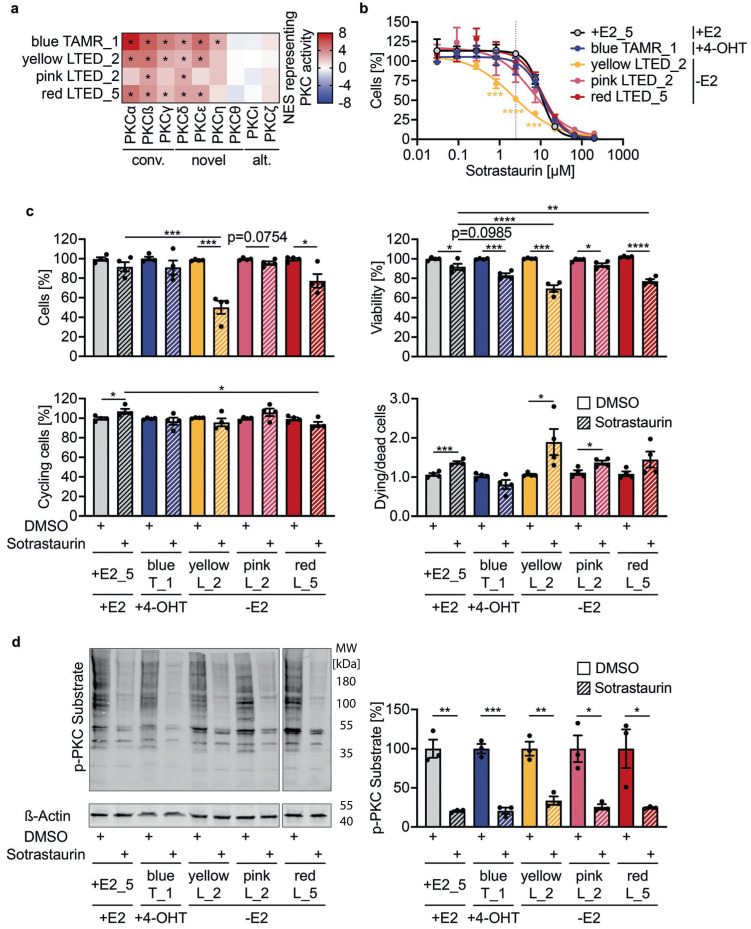
PKC is a potentially shared contributor to endocrine therapy resistance. **a** Relative kinase activities of conventional (conv.), novel, and atypical (alt.) PKC isoforms were determined using phosphoproteomic data. NES representing activation in MCF7 TAMR and LTED clones compared to the +E2 control is presented. Significant activation (absolute scores ≥ 2) is indicated by asterisks. **b** Sensitivity testing to the pan-PKC inhibitor Sotrastaurin. Indicated MCF7 clones, and +E_5 replicate, were kept in the specified media (+E2, +4-OHT, -E2) and treated with Sotrastaurin. Cell numbers were determined by nuclei counting at day 0 and day 7 of culture with indicated treatments and normalized to the DMSO controls (*n* = 4 with 3 technical replicates each). Gray dotted line indicates effect at 2.50 µM Sotrastaurin. Signifiance *p < 0.05, **p < 0.01, ***p < 0.001 as determined by two-way ANOVA with Dunnett multiple comparisons test. **c** Clones were treated with DMSO (solid filling) or 2.50 µM Sotrastaurin (shaded filling). Nuclear cell count and ATP levels were determined 6 days after treatment start, while EdU and DAPI incorporation were assessed 4 days after treatment start. Results were normalized to DMSO controls. *n* = 4 with ≥4 technical replicates each. Shown are mean ± SEM. *p < 0.05, **p < 0.01, ***p < 0.001, ****p < 0.0001 as determined by unpaired two-tailed *t*-tests (DMSO vs. Sotrastaurin treatment) or one-way ANOVA with Dunnett multiple comparisons test (Sotrastaurin treated conditions). **d** Clones were treated with DMSO or Sotrastaurin for 24 h, stimulated with 200 nM TPA for 30 min, and harvested. Representative Western Blot and quantification of three biological replicates of MCF7 cells treated with DMSO (solid filling) or 2.50 µM Sotrastaurin (shaded filling) for 24 h before stimulation. Shown are mean ± SEM. *p < 0.05, **p < 0.01, ***p < 0.001 as determined by unpaired two-tailed *t*-tests.

Kinase activity analysis revealed significantly higher activation of several isoforms of protein kinase C in MCF7 TAMR as well as in LTED clones compared to the +E2 control replicates (Fig. 6a). Treatment of the cell lines with the pan-PKC inhibitor Sotrastaurin showed higher sensitivity of the *yellow* MCF7 LTED_2 clone (Fig. 6b) compared to the other clones and replicates. Treatment with 2.50 µM Sotrastaurin reduced the proliferative capacity of these cells by almost 60% compared to +E2_5 cells (Fig. 6c). Accordingly, viability and cell death were reduced and enhanced, respectively, while the number of cycling cells was not affected. This suggests that the inhibitory effect of Sotrastaurin was mostly conferred by increased induction of cell death. Cellular viability was significantly reduced also in MCF7 *blue* TAMR_1 and *red* LTED_5 clones upon treatment with 2.50 µM Sotrastaurin and showed a trend when compared to Sotrastaurin-treated +E2_5 control cells (Fig. 6c). Yet, activation of PKC by TPA was significantly and similarly repressed by 66–80% in all endocrine sensitive and resistant clones tested (Fig. 6d), pointing at a similar inhibition of PKC signaling. The consistent effects in reduced cellular viability in MCF7 *yellow* LTED_2 and to a lesser extent also for *red* LTED_5 clones, thus point to PKC activation as a potentially shared contributor to endocrine therapy resistance.

### Mechanisms contributing to clonal endocrine resistance have potential clinical relevance

**Fig. 7:**
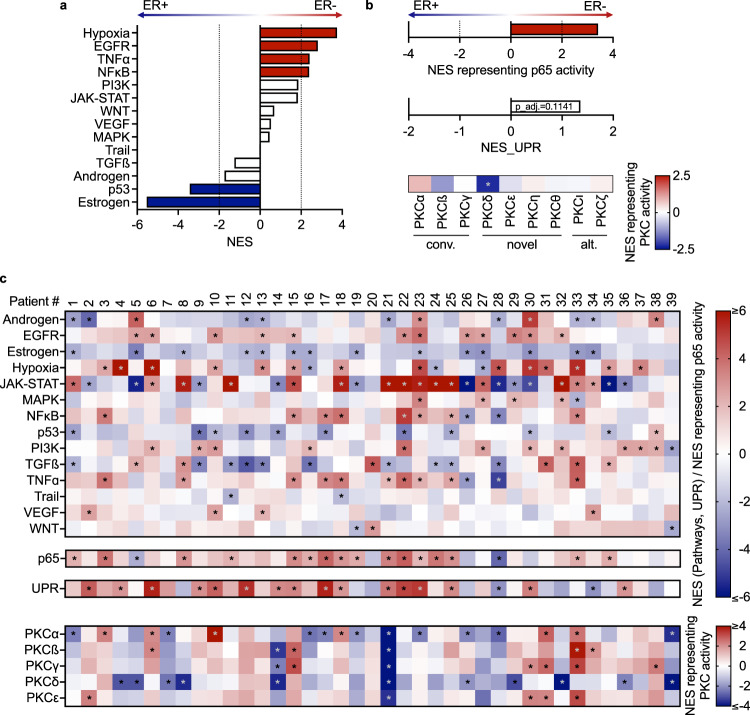
Activation of UPR and PKC is reflected in breast cancer patients from the CPTAC cohort. Patients in the CPTAC-BRCA cohort^21^ were stratified into patients with ER− (*n* = 39) and ER+ (*n* = 81) disease. RNA-Seq data from these patients was utilized to infer **a** pathway activityies^25,26^, **b** activity of p65^30,31^ and UPR^57^. The adj. *p*-value is given for UPR activation. Phosphoproteomic data from these patients was utilized to determine the activity of conventional (α, β, γ) and novel (δ, ε) PKC isoforms. The asterisk indicates significant repression (of PKCδ) in patients with ER− disease. **c** Same analysis as in **a** and **b**, however, on a per patient basis. Pathway (upper), p65 (second) and UPR activation (third panel) as assed by comparing z-scored RNA-seq data between individual patients with ER− disease to the total reference cohort. PKC activity estimates (lower panel) were based on the common reference sample as described by ref. ^21^. Significant relative activation and repression are indicated by asterisks. For UPR activation, absolute NES ≥ 2 and concurrent adj. *p* < 0.05 were considered significant.

Finally, we assessed clinical relevance of our in vitro findings. To this end, we retrieved and analyzed data from the CPTAC-BRCA cohort^21^, which contains paired gene expression and phospho-proteomic data from 120 treatment-naïve breast cancer patients with known ER−status, i.e., ER− (*n* = 39) and ER+ (*n* = 81) disease. Here, we were focused on patients with ER− disease; tamoxifen treatment and estrogen deprivation had abrogated estrogen signaling in our in vitro models, rendering them ‘quasi’ ER−, and ‘subtype switching’ has been reported for luminal A and luminal B patients upon endocrine therapy^36^. Pathway analysis of the CPTAC RNA-seq data showed significantly elevated NFκB signaling in patients of the ER− subtype compared to patients with ER+ disease (Fig. 7a), in line with the observations we made in all MCF7 *blue* TAMR_1 and LTED clones. Similarly, TNFα, JAK-STAT, and hypoxia signaling were associated with ER- disease, also matching our in vitro findings in MCF7. Further, p65 activity was significantly associated with ER− patients, while the UPR showed only a trend. Different from our in vitro models, activation of different PKC isoforms was not associated with either ER− or ER+ disease in the patients (Fig. 7b). We thus performed data analysis on a per patient basis to correlate our in vitro results with alterations in individual patients within a potentially heterogeneous cohort.

Indeed, patient-specific pathway activity analysis highlighted strong inter-patient heterogeneity in both the ER− (Fig. 7c) and the ER+ cohorts (Supplementary Fig. 7a–d). JAK-STAT signaling was significantly activated and downregulated in 15 and 12 patients with ER− disease, respectively. This heterogeneity is consistent with our in vitro models, where we had observed upregulation of JAK-STAT signaling only in the MCF7 LTED clones while this pathway was not activated in the *blue* TAMR_1 clones (compare Fig. 4a). Significant activation and repression of p65 activity was evident in 16 and 2 patients with ER− disease, respectively (Fig. 7c). Similarly, 16 and 2 patients with ER- disease showed significant activation and repression of UPR, respectively. Inter-patient heterogeneity was, however, observed also in ER+ disease. There, 6 patients showed significant up- and another 19 downregulation of p65 activity (Supplementary Fig. 7b), while 9 and 27 patients had activated and repressed UPR, respectively (Supplementary Fig. 7c). Finally, we investigated PKC activity at the single patient level and indeed observed strong inter-patient heterogeneity (lower panel in Fig. 7c). Two ER− patients (#31 and #33) showed consistent activation of PKCα, PKCβ, PKCγ, and PKCε. The same kinases were upregulated also in several other patients with ER− disease, yet not reaching significance. In contrast, PKCδ was hardly activated in any patients compared with the reference sample utilized by Krug et al.^21^ and was significantly activated in only four out of the 120 CPTAC patients, all with ER+ disease (Supplementary Fig. 7d). In contrast, we found activation of PKCδ in all MCF7 TAMR and LTED clones, however, this did not correlate with sensitivity to Sotrastaurin. PKCδ might thus not be the main determinant of Sotrastaurin sensitivity in our cell line models which is corroborated by the observation that the activities of the other PKC isoforms in several ER− patients were consistent with our in vitro models. It is tempting to speculate that these patients might benefit from (pan-) kinase inhibitors of PKC. Intriguingly, all PKC activities, also of PKCδ, were downregulated in some patients (e.g., #21), further supporting the need to assess pathway activities at the single patient level. Taken together, our in vitro models were in agreement with clinical findings from the CPTAC-BRCA cohort and particularly with ER−negative disease.

## Discussion

Endocrine therapy resistance remains an urgent clinical problem as up to 41% of patients aged younger than 75 years who originally presented with advanced disease (i.e., pT2N ≥ 2: tumor diameter >2 cm and at least 4–9 positive lymph nodes) relapse within 20 years after initial diagnosis and treatment start^37^. Recent findings have highlighted the importance of tumor heterogeneity in disease progression^11,38^. In our study we set out to answer two questions: First, we aimed at deciphering the contribution of primary and secondary resistance to commonly applied first line endocrine therapeutics, namely Tamoxifen and aromatase inhibitors. Second, we wanted to unravel clinically relevant contributors to endocrine therapy resistance in individual clones. To this end, we (i) utilized a bulk barcoding approach in two luminal breast cancer cell line models, (ii) isolated individual clones from complex cell pools, and (iii) validated private and potentially shared contributors to endocrine therapy resistance. We are aware of some limitations our study has as we employed tumor cell lines and did not perform in-depth genetic characterization of the clones.

Our bulk analysis showed that Tamoxifen resistance was achieved by either selection of primary resistant clones or acquisition of secondary resistance. Recurrent outgrowth of clones carrying identical barcodes was prominent in the T47D TAMR and LTED models suggesting primary resistance in these cell line models. In contrast, the lack of commonly selected barcodes in any MCF7 replicates pointed at the acquisition of secondary resistance as the likely mechanism there. The latter observation is not in line with ref. ^18^, who had shown predominant selection of primary resistant clones to Tamoxifen and the estrogen degrader Fulvestrant. In our LTED models, we observed a combination of likely primary (e.g., T47D *orange* LTEDs) as well as secondary resistance (e.g., MCF7 *red* LTED_5 and T47D *brown* LTED_2). In all cell line models, different clones were selected in TAMR vs. LTED conditions, indicating independent modes of resistance to the two treatment regimes.

In the T47D model, the dominance of *orange* barcoded cells in the untreated +E2_5 replicate suggested a fitness advantage, like stronger proliferation, over other clones in the same replicate. When analyzing single clones, however, *green* + E2_5 clones proliferated significantly stronger than their *orange* counterparts. These findings point at clonal interactions, cooperativity and competition^39^, which is likely to influence dynamics of different clones and that is lost when cultures are grown at the single clone level.

Initial analysis of differential pathway activities based on the RNA-Seq data highlighted strong inter- and intra-tumor heterogeneity, as estrogen signaling was the only significantly suppressed pathway in all isolated T47D and MCF7 TAMR clones. RNA-Seq revealed activation of the UPR as a private event for MCF7 TAMR clones. In line, elevated expression of a UPR gene signature was previously found to correlate with worse relapse-free survival during Tamoxifen treatment^40^. Conversely, proteasome inhibitors such as Bortezomib have been hypothesized to potentiate and induce a detrimental UPR^35^. Accordingly, we observed strong sensitivity to Bortezomib for *blue* TAMR_1 cells, which showed an activation of the UPR.

While UPR activation was a private event in our models, activation of PKC was observed in all MCF7 TAMR and LTED clones. Treatment with 2.50 µM Sotrastaurin preferentially reduced the cellular viability of *yellow* MCF7 LTED_2 clones and showed a trend also for *red* LTED_5 clones. Accordingly, different PKC isoforms have previously been implicated in endocrine therapy resistance and/or estrogen independent growth, such as PKCα^41^ and PKCε^42^. Mechanistically, we found that treatment with 2.50 µM Sotrastaurin did not significantly impact cells passing S-phase, which is in line with recent findings^43^, but rather induced cell death.

The analysis of patient data from the CPTAC-BRCA cohort supported the relevance of our in vitro findings, and the heterogeneity of breast tumors at pathway, kinase and transcription factor activity levels. JAK-STAT, p65, and UPR activation was evident in a number of individual patients with ER− disease corroborating our in vitro results. However, activation of PKCα, PKCβ, PKCγ and PKCε was observed in just two patients with ER− disease (#31 and #33) and resembled the activity patterns in MCF7 TAMR and LTED clones. Our data suggests that patients with activation in UPR or PKC might benefit from treatment with the proteasome inhibitor Bortezomib or the PKC inhibitor Sotrastaurin in respective first line therapies. Multi-omics analysis, including that of phosphoproteomic data, turned out as essential to fully capture the heterogeneity in vitro and in patients, supporting the use of proteomics in precision oncology^44^.

## Methods

### Generation of barcoded endocrine resistant cell lines

MCF7 (Cellosaurus CVCL_0031) and T47D (CVCL_0553) were obtained from ATCC (LGC Standards GmbH, Wesel, Germany). Cells were kept at 37 °C with 5% CO2 in a humidified atmosphere in Dulbecco’s Modified Eagle Medium (DMEM) supplemented with 10% FCS, 50 units/ml penicillin and 50 g/ml streptomycin sulfate (Invitrogen AG, Carlsbad, CA, USA), and 10^-8^M 17-ß-estradiol (E2, Sigma-Aldrich, Saint-Louis, MI, USA). The ClonTracer library^22^ (Addgene # 67267) was co-transfected with second generation viral packaging plasmids VSV.G (Addgene #14888), and psPAX2 (Addgene #12260) into HEK293FT (Cellosaurus CVCL_6911) obtained from Thermo Fisher Scientific (Waltham, MA, USA). 48 h after transfection, the supernatant was cleared by centrifugation (500 × *g*/5 min) and passed through a 0.45 µm filter. 1 × 10^5^ MCF7 and T47D cells were transduced with the viral particles in the presence of 10 µg/mL Polybrene (Merck, Darmstadt, Germany) at a multiplicity of infection (MOI) of 0.05. Generation of viral particles and the transduction of T47D and MCF7 were performed by the Cellular Tools Core Facility, German Cancer Research Center (DKFZ). 1 µg/mL Puromycin (Merck) was used to select for successful integration events and transduced cells were initially expanded. Five replicates each were either frozen down immediately, kept under control conditions (+E2), were treated with 100 nM 4-hydroxytamoxifen (4-OHT, Sigma-Aldrich) i.e., the biologically more active metabolite of Tamoxifen^45^, or were deprived of E2 and therefore kept in DMEM (w/o phenol red) supplemented with 10% charcoal stripped FCS. Continuous exposure for 8 months to either endocrine treatment rendered the barcoded cells Tamoxifen-resistant (TAMR) or resistant to long-term estrogen deprivation (LTED). During this time, cells were passaged when they had reached 80% confluency or reseeded in a new dish if they had not reached this within 4 weeks. Medium was changed every week to ensure availability of nutrients and to replenish the respective treatment, i.e., +E2 with +E2 media, TAMR with +4-OHT media, and LTED with -E2 media, except when otherwise stated. Control cells (+E2) required passaging on average every 4 days throughout. All cell lines were regularly authenticated by STR profiling (Multiplexion GmbH Heidelberg, Germany) as well as tested for potential mycoplasma contamination.

### Barcode composition analysis

DNA was isolated using the DNeasy Blood & Tissue kit (Qiagen, Hilden, Germany) according to the manufacturer’s instructions. The barcode region was amplified as described (https://www.addgene.org/pooled-library/clontracer/, accessed 22/12/12) with the adapted thermocycling program: 95 °C–5 min, 30x (95 °C–30 s, 69 °C – 15 s, 72 °C – 9 s) and 72 °C – 7 min. PCR products were cleaned with the Wizard® SV Gel and PCR Clean-Up System (Promega, Madison, WI, USA). Concentration and size were determined with Bioanalyzer 10 (Agilent, Santa Clara, CA, USA). Ten samples were multiplexed per lane and sequenced on the HiSeq 2000 V4 Single-read 50 bp with the custom sequencing primer: 5′-TCTACACACTGACTGCAGTCTGAGTCTGACAG-3′. Sequencing was performed by the DKFZ NGS Core Facility. Barcode analysis was performed as described (https://github.com/luca8651/Barcode_analyses-python, accessed: 22/12/07). Barcodes with at least 5 × 10^5^ combined reads in all initial, +E2, TAMR and LTED replicates for each cell line (MCF7 or T47D) were log2 normalized and depicted as violin plots using ggplot2 (version 3.4.1) in R (version 4.0.2).

### Isolation of clonal endocrine resistant subpopulations

Eight barcoded treatment naïve or resistant MCF7 or T47D cell pools were used to isolate clonal subpopulations utilizing a Cytena single cell spotter (https://www.cytena.com/wp-content/uploads/2022/07/F.SIGHT-Deconvoluting-clonal-complexity-of-barcoded-cell-populations-Rainar-Will-2022.pdf) or manual dispension. Clonality was verified by assessment of images taken during spotting of single cells, and by Sanger sequencing of barcode regions once clones had grown out. For the latter, DNA and RNA was isolated using the AllPrep DNA/RNA Micro Kit (Qiagen) according to the manufacturer’s instructions. Concentrations were determined with a NanoDrop® ND-1000 UV-Vis Spectrophotometer (Thermo Fisher Scientific). Barcode regions were amplified using Phusion Hot Start II DNA-Polymerase (2 U/µL) (Thermo Fisher Scientific) according to the manufacturer’s instructions using the following primers: Fw: 5′-GCTGTGCCTTGGAATGCTAGTTGG-3′ and Rev: 5′-TCTGCTGTCCCTGTAATAAACCCG-3′. Thermocycling was performed as: 98 °C – 2 min, 32x (98 °C – 10 s, 71 °C – 20 s, 72 °C – 70 s) and 72 °C – 10 min. PCR clean-up and Sanger sequencing were performed by Eurofins Genomics Germany GmbH (Ebersberg, Germany). Sequences were analyzed using SnapGene software (Dotmatics, Bishop’s Stortford, UK). Sanger chromatograms of some MCF7 TAMR and LTED clones were too complex for unambiguous determination of barcodes, thus necessitating deep sequenced using Illumina MiSeq technology. To this end, published reverse primers (https://www.addgene.org/pooled-library/clontracer/, accessed: 22/12/12) were extended by three bases (CTG) at the 3′ end. PCR amplification was performed for 48 isolated clones with Phusion Hot Start II DNA-Polymerase (2 U/µL) according to the manufacturer’s instructions and thermocycling was performed as: 98 °C–2 min, 32x (98 °C–10 s, 69 °C–20 s, 72 °C–12 s) and 72 °C–10 min. PCR products were cleaned using the Wizard SV Gel and PCR Clean-up System (Promega) according to manufacturer’s instructions. Concentration and size were evaluated with the Agilent 2200 TapeStation System (Agilent). Samples were sequenced as single library on the MiSeq V2 300 Nano (Illumina, San Diego, CA, USA) with the custom sequencing primer: 5′-TCTACACACTGACTGCAGTCTGAGTCTGACAG-3′. Sequencing was performed by the DKFZ NGS Core Facility. Barcodes of interest were identified in MiSeq derived fastq files using a custom script. Using R (version 4.0.3)^46^ and R studio^47^, the ShortRead package^48^ was used to read fastq files individually. For each of the files, occurrence of the respective barcodes in the DNA sequence was counted searching for the respective sequence. For MCF7 TAMR_1 clones, highly similar reads were manually clustered and violin plots generated in GraphPad Prism (version 9.4.1; GraphPad, Boston, MA, USA). One outlier was identified using the ROUT method (Q = 0.1%) and removed from the data.

### Integration site analysis

Virus integration sites were identified in MCF7 + E2_3 and LTED_5 as well as T47D + E2_3, TAMR_2 and LTED_2 by Genewerk GmbH (Heidelberg, Germany) as previously described^23^. The top predicted integration sites of individual complex barcoded cell pools were validated by a PCR-based assay using the Phusion Hot Start II DNA-Polymerase (2 U/µL) (Thermo Fisher Scientific) according to manufacturer’s instructions. To this end, a primer binding only to the viral 3′LTR with the sequence 5′-CCCAACGAAGATAAGATCTGC-3′ was combined with primers binding adjacent to the identified individual integration sites. Consequently, integration sites validated in the complex barcoded cell pools were investigated in isolated clones. Thermocycling was performed as: 98 °C–2 min, 32x (98 °C–10 s, 65 °C–20 s, 72 °C–60 s) and 72 °C–10 min. PCR products were subjected to Sanger sequencing (Eurofins Genomics) and sequences analyzed with SnapGene software.

### Assessment of ESR1 hot-spot mutation status in individual clones

Sequence covering the hot spot mutation site (codon 536–538) in the *ESR1* gene was amplified from single clones representing each of the isolated populations using Phusion Hot Start II DNA-Polymerase (2 U/µL) according to manufacturer’s instructions and the following primer pair: Fw: 5′-AATACCCACTCCTGCTTGGC-3′, Rev: 5′-TATCTGAACCGTGTGGGAGC-3′. Thermocycling was performed as: 98 °C–2 min, 32x (98 °C–10 s, 66 °C–20 s, 72 °C–60 s) and 72 °C–10 min. PCR products were subjected to Sanger sequencing (Eurofins Genomics) and sequences were analyzed with SnapGene software.

### Analysis of cell proliferation and viability

Cells were plated in black clear-bottomed 96 well plates (Greiner Bio-One International GmbH, Kremsmünster, Austria). Cell numbers were assessed either by counting RFP-positive cells or counting of nuclei after Hoechst 33342 staining using a IXM XLS microscope (Molecular Devices, San Jose, CA, USA). Cells were detected and counted with Molecular Devices Software using default settings for minimum RFP intensity, size of nuclei, and staining intensity. Cell numbers were expressed relative to the seeding or treatment controls. Cell viability was determined using the CellTiter-Glo® Luminescent Cell Viability Assay (Promega) according to the manufacturer’s recommendation. Luminescence was measured using a GloMax Discover System (Promega).

### Analysis of cycling and dying/dead cells

Cells in S-phase were determined using the Click-iT™ EdU Cell Proliferation Kit for Imaging with Alexa Fluor™ 594 dye (Thermo Fisher Scientific) following the manufacturer’s recommendation, however, adapted to 96 well format. Cells were plated in black clear-bottomed 96 well plates and pulsed for 21 h with 10 µM EdU 72 h post treatment start or transfection by replacing half of the media with fresh growth media containing 20 µM EdU. Cells in S-phase were determined as Alexa Fluor 594 positive and normalized to total cell numbers as determined by Hoechst 33342 staining using a IXM XLS microscope. Dying/dead cells were stained with 1 µg/mL DAPI 96 h post treatment start or transfection, and counted with a IXM XLS microscope. Total cell numbers were determined by Hoechst 33342 staining and ratios were calculated.

### Inhibitor treatment and siRNA transfection

Cells were either treated with the proteasome inhibitor Bortezomib^49^ or the pan-PKC inhibitor Sotrastaurin^50^. Inhibitors were purchased from MedChemExpress (Monmouth Junction, NJ, USA) and resuspended in dimethyl sulfoxide (DMSO, Sigma-Aldrich). Resulting data were normalized to DMSO controls. A pool of four siRNAs targeting *RELA* (LQ-003533-00-0002) and a non-targeting control (D-001810-10-20) were obtained from Dharmacon (Lafayette, CO, USA) and cells were transfected using RNAiMAX® (Thermo Fisher Scientific) in media lacking penicillin and streptomycin.

### RNA-Sequencing

RNA was isolated from clonal cell line populations using the AllPrep DNA/RNA Micro Kit (Qiagen) according to the manufacturer’s instructions. RNA-Sequencing (RNA-Seq) was performed by the DKFZ NGS Core Facility using NovaSeq 6 K PE 50 SP for MCF7 and NovaSeq 6 K PE 50 S1 for T47D clones (Illumina). Initial data QC of raw sequence data was done using FASTQC (https://www.bioinformatics.babraham.ac.uk/projects/fastqc/, version 0.11.9). Alignment of reads to the human reference genome (“GRCh38.p13.primary_assembly_gencode” (https://www.gencodegenes.org/human/release_39.html) was done using the Rsubread R package (version 2.6.4)^51^. Gene expression counts were quantified using the featureCounts pipeline^52^ with default settings, implemented also in the Rsubread R package. Initial processing, alignment and quantification were performed by the Omics IT and Data Management Core Facility (ODCF) using the DKFZ Compute Cluster running under CentOS Linux 7 (Core) (https://odcf.dkfz.de/). Complete downstream RNA-Seq analysis was performed with R (R version 4.1.0)/Bioconductor software^53^ separately for each cell line, using the same settings. Initially, a non-specific intensity procedure was applied (function filterByExpr::edgeR R package) to remove non-expressed genes (<10 reads in at least one of the barcoding clone biological conditions). Normalization of the remaining gene counts was performed using TMM (Trimmed Mean of *M*-values) from edgeR R package (version 3.36.0)^54^, followed by the voom function from the limma R package (version 3.50.0), aiming to increase statistical power and to account for non-biological variability^55^. Finally, differential expression analysis between the different cell lines and clones was performed using the limma R package^56^.

### Pathway and TF activity analysis

PROGENy (Pathway RespOnsive GENes) R package (version 1.16.0) was utilized to disentangle transcriptional heterogeneity related to differentially activated pathways. PROGENy infers the activity of 14 major signaling pathways^25,26^. Differentially activated transcriptional factors were retrieved using the decoupleR R package (version 2.1.6) to implement robust statistical models^31^. Then, the DoRothEA gene regulatory network resource R package (version 1.6.0) was applied to predict transcription factor (TF)—target gene interactions (A to C confidence levels)^30^.

### Functional enrichment analysis

Gene Set Enrichment Analysis (GSEA) was applied using the clusterProfiler R package (version 4.2.2)^57^ and MsigDB All Hallmarks(H) gene sets from the msigdbr R package (version 7.5.1) (https://github.com/DavisLaboratory/msigdb) using default settings.

### RT-qPCR

cDNA was synthesized from the isolated RNA using the RevertAid RT Reverse Transcription Kit according to the manufacturer’s instructions. Quantitative Reverse Transcription-PCR (RT-qPCR) was performed using the Power SYBR Green PCR Master Mix (2x) (Thermo Fisher Scientific) and a QuantStudio™ 5 Real-Time PCR System (Thermo Fisher Scientific). Data analysis was performed using QuantStudio™ Design & Analysis Software v1.5.0. Relative changes were calculated by the comparative Ct method (ΔΔCt method)^58^ and relative expressions were visualized as 2^−ΔΔCt^. *ACTB* forward: 5′-ATTGGCAATGAGCGGTTC-3′, *ACTB* reverse: 5′-GGATGCCACAGGACTCCA-3′, *GAPDH* forward: 5′-GAGTCCACTGGCGTCTTCAC-3′, *GAPDH* reverse: 5′-GTTCACACCCATGACGAACA-3′, *RELA* forward: 5′-GCTTGTAGGAAAGGACTGCC-3′, *RELA* reverse: 5′-GCTGCTCTTCTATAGGAACTTGG-3′.

### Mass spectrometry

Cells were rinsed with ice-cold PBS and lysed in RIPA buffer (Thermo Fisher Scientific) supplemented with 1x cOmplete EDTA-free protease inhibitor, 1x PhosSTOP phosphatase inhibitor (both Roche, Basel, Switzerland), 10 mM NaF (Bernd Kraft, Duisburg, Germany), 1 mM Na3VO4 (Sigma-Aldrich), 250 U/ml Benzonase (Merck) and 10 U/mL Rnase-Free Dnase (Qiagen). Lysates were incubated on ice for 30 min and cleared by centrifugation. Protein concentrations were determined using the Pierce™ BCA Protein Assay Kit (Thermo Fisher Scientific) according to the manufacturer’s instructions.

Proteins (320 µg protein/sample) were precipitated^59^. Afterward, the protein pellet was digested first using Lysyl endopeptidase for 4 h (Lys-C, Fujifilm, Hong Kong, China) and then using trypsin for 16 h, both at 37 °C. Peptides were desalted using Sep-Pak C18 cartridges (Waters, Milford, MA, USA) and an Immobilized Metal Affinity Chromatography (IMAC) column (Thermo Fisher Scientific) charged with FeCl_3_ was used for the enrichment of phosphorylated peptides^60^. The fraction containing the phosphopeptides was collected, and vacuum centrifuged to dryness. Enriched phosphopeptides were dissolved in 0.1% TFA and desalted using Stop and Go Extraction tips (Supelco, Bellefonte, PA, USA)^61^. Each sample was analyzed via LC-MS/MS for a total of 120 min of analysis time. Peptides were separated according to their hydrophobicity by liquid chromatography (UltiMate 3000, Thermo Fisher Scientific) with a 102 min linear gradient of 2–28% acetonitrile (Waters, 186008795, BEH C18 130 Å 1.7 µm 75 × 250 mm). The LC system was directly coupled to a MS system (Orbitrap Exploris 480, Thermo Fisher Scientific) via electrospray ionization. MS analysis was performed using data-independent acquisition (DIA). MS1 scans were acquired at a resolution of 120 K covering the range from 350–1400 m/z. Maximum injection time was 45 ms and the automated gain control (AGC) target was set to 3e6. MS2 acquisition was performed using 48 precursor isolation windows of variable width and 1 m/z overlap that covered the range from 400–1200 m/z. Fragment spectra were acquired at a resolution of 30 K and a normalized collision energy of 26% was applied. Maximum injection time was 54 ms and the AGC target was set to 1e6.

Peptide and protein identification and quantification from DIA raw data was performed with directDIA in the Spectronaut software (version 15, Biognosys, Switzerland) using the built in identification and localization algorithm^62^. The search was performed against the human proteome fasta file (Uniprot, downloaded on July 14th, 2020, with 74,811 entries). Default settings were applied with phosphorylation (S,T,Y) as an additional variable modification (localization cut-off = 0). Identified phosphorylated peptides were site-collapsed using the Perseus^63^ (version 1.6.2.3) plug-in PeptideCollapse^62^ and a localization cut-off at 0.95 was applied.

### Phosphoproteomics data analysis

Raw data output from Perseus was initially processed using the PhosR R package (version 1.4.0)^64^. Raw phosphosite intensities were normalized using variance stabilizing transformation from the vsn R package (version 3.62.0)^65^. In addition, phosphosites were removed if they were not quantified in at least 2 out of 3 replicate samples in at least one condition. Differential phosphosite abundance analysis was performed with the limma R package implementing the limma-trend pipeline^56^.

Differentially activated kinases were identified initially utilizing the OmniPath R package (version 3.2.5) to retrieve enzyme-PTM relationships, in form of molecular networks with directed interactions and effect signs^32^. Then, the decoupleR R package (version 2.1.6) was used to implement specific statistical models (normalized weighted mean) to infer kinase activities^31^. The estimated activity score for each kinase was approximated by weighting the molecular readouts of its targets by their *mode of regulation* (Activation or Inhibition) & their relative *likelihood*. Only kinases having at least 5 target proteins (phosphosite_target) present in the analyzed phosphoproteomics data were regarded.

### Western blotting

Proteins were separated by SDS-PAGE^66^ using PageRuler™ Prestained Protein Ladder (Thermo Fisher Scientific) as size maker. Afterwards, proteins were blotted using Trans-Blot Turbo™ Mini PVDF Transfer Packs and the Trans-Blot Turbo™ Transfer System (Bio-Rad Laboratories, Hercules, CA, US) according to the manufacturer’s instructions. Membranes were blocked in Blocking Buffer for Fluorescent Western Blotting (Rockland, Philadelphia, PA, USA): TBS supplemented with 10 mM NaF and 1 mM Na_3_VO_4_. Subsequently, the membranes were incubated with antibodies targeting ß-Actin (0869100-CF, MP Biologicals, Irvine, CA, USA, 1:1500 dilution), p65 (CST8242, Cell Signaling Technology, Danvers, MA, USA, 1:1000 dilution), and p(Ser) PKC Substrate (CST2261, 1:1000 dilution) in blocking buffer at 4 °C overnight. The next day, membranes were washed three times for 5 min each with TBS containing 0.1% Tween 20 (TBS-T, Merck), incubated with anti-mouse (SA5-35521, Thermo Fisher Scientific) or anti-rabbit antibodies (A-21077, Thermo Fisher Scientific) and washed again. Finally, (phosphorylated) proteins were visualized with Odyssey Infrared Imaging Software and quantification was performed using Image Studio Lite (LI-COR, Lincoln, NE, USA). Uncropped images of respective Western blots are shown in Supplementary Information.

### Clinical dataset

Raw data from patient-derived gene expression and phospho-proteomics data of the CPTAC-BRCA^21^ clinical dataset were obtained from the cBioPortal open-source database^67,68^. Initial data analysis was done as described for the in vitro data above. The CPTAC-BRCA cohort was subdivided for primary analysis into patients with ER− (*n* = 39) and ER+ disease (*n* = 81). Pathway and transcriptional factor activities were estimated also per patient basis, using as an input the scaled and normalized gene expression values from cBioPortal. To this end, Z-score transformation was applied prior to per patient analysis, to uncover higher or lower activities within individual patients compared to the total reference cohort. Single sample gene set enrichment analysis (https://github.com/broadinstitute/ssGSEA2.0)^69^ was applied on the scaled gene expression values using *Z*-score transformation as above. The script “ssgsea-gui.R” was applied with default settings (sample.norm.type: none, weight: 0.75, statistic: area.under.RES, nperm: 1000, output.score.type: NES and correl.type: z.score) and as gene-set library the included hallmark signatures file (version 7.5.0) in gmt format was selected (https://github.com/broadinstitute/ssGSEA2.0/tree/master/db/msigdb).

### Reporting summary

Further information on research design is available in the Nature Research Reporting Summary linked to this article.

### Supplementary information

Supplementary Information

nr-reporting-summary

## Data Availability

The RNA sequencing data of the clonal and bulk MCF7 and T47D cell line samples generated in this study have been deposited in the European Genome-Phenome Archive under the accession number EGAS00001007123. The data are available under controlled access due to the sensitive nature of human sequencing data, and access can be obtained by contacting the appropriate Data Access Committee listed in the study for each dataset. Access will be granted to commercial and non-commercial parties according to patient consent forms and data transfer agreements. We have an institutional process in place to deal with requests for data transfer. A response to requests for data access can be expected within 14 days. After access has been granted, the data is available for two years. The mass spectrometry phospho-proteomics data from clonal and bulk cell line samples have been deposited to the ProteomeXchange Consortium via the PRIDE^70^ partner repository with the dataset identifier PXD040478. The uncropped Western blot images shown in Fig. 6d and Supplementary Fig. S6c are provided in the Source Data file with this paper. The remaining data are available within the article, Supplementary Information, Source Data file, Zenodo (10.5281/zenodo.7780930) or github (Jasonmbg/BRCA_Clonal_Resist_Project and Jasonmbg/PublicData.CPTAC.BreastCancer.PhosphoP).
