# Supplementary material for: Clonal heterogeneity in ER+ breast cancer reveals the proteasome and PKC as potential therapeutic targets
Source: NPJ Breast Cancer. 2023 Dec 2;9:97. doi: 10.1038/s41523-023-00604-4 (PMC10693625; doi:10.1038/s41523-023-00604-4)
Supplement: Supplementary file 1 — Supplementary Information [file 41523_2023_604_MOESM1_ESM.pdf]

Table of contents

Supplementary Figures cited in the manuscript.. 2

Supplementary Tables 16

Supplementary Figures: Uncropped scans of Western blots 20

Supplementary References 22

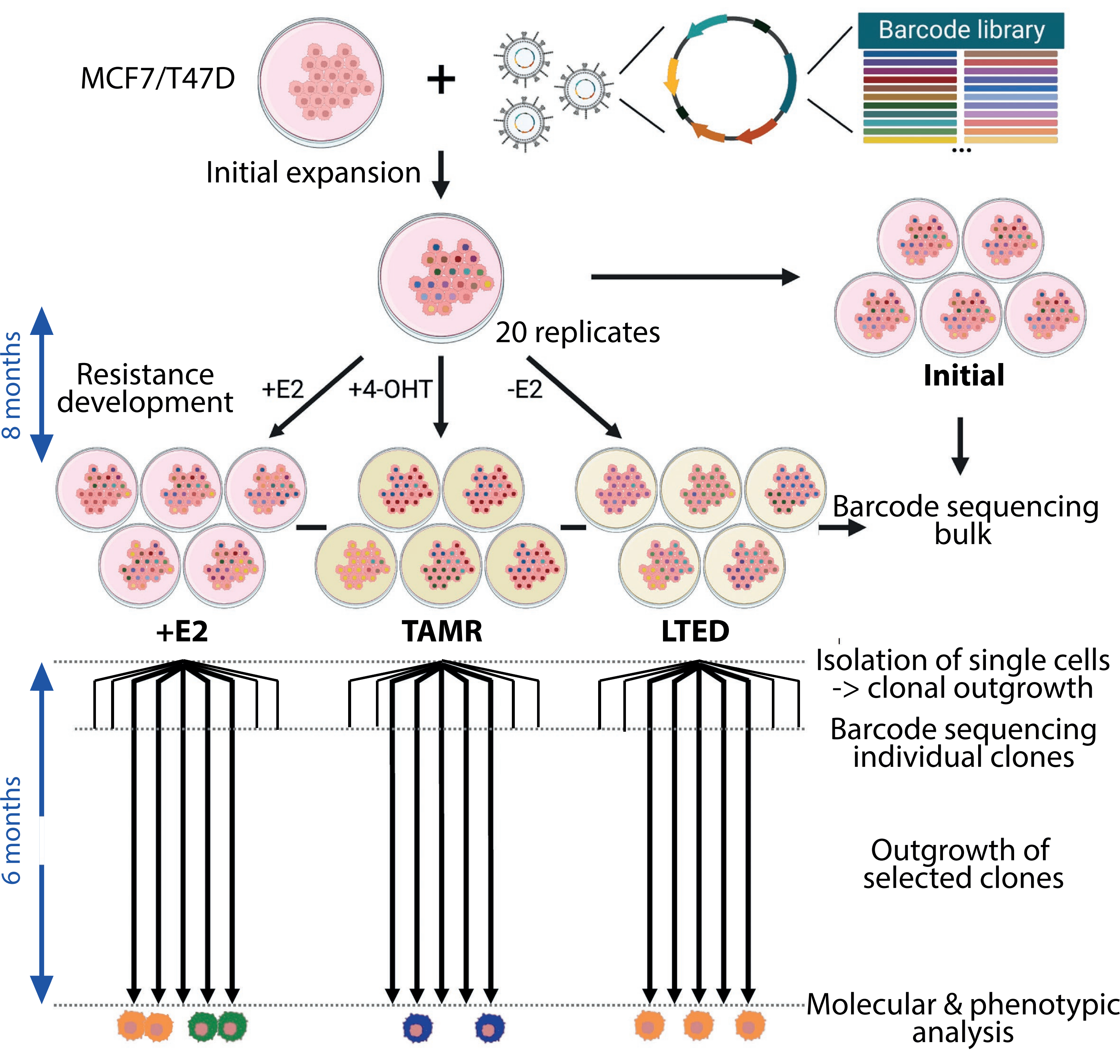
MCF7/T47D
+
Barcode library
...
Initial expansion
20 replicates
Initial
8 months
Resistance development
+E2
+4-OHT
-E2
Barcode sequencing bulk
+E2
TAMR
LTED
Isolation of single cells -> clonal outgrowth
Barcode sequencing individual clones
6 months
Outgrowth of selected clones
Molecular & phenotypic analysis

Supplementary Figure 1: Outline of study. MCF7 and T47D cells were transduced with the ClonTracer library [1] at an MOI of 0.05. Transduced cells were expanded under purmocyin selection. Then, the culture was split into 20 replicates. Five biological replicates (Initial) were frozen to later assess the initial barcode-complexity in the bulk populations. Fifteen replicates were cultured in three different treatment conditions with five biological replicates each: One set of replicates was kept under control growth conditions (+E2), another was treated with 4-hydroxytamoxifen (4-OHT) the third was deprived of estrogen (-E2). Respective treatments were maintained for eight months to generate control, Tamoxifen resistant (TAMR), and long-term estrogen deprived (LTED) cell lines. Bulk barcode sequencing was performed with all replicates. Then, single cells were isolated from selected replicates, grown out, and barcodes in these clones were sequenced. Culturing of selected clones was continued until sufficient material for molecular and phenotpic analysis was available, which took at least six months from the time single cells had been isolated. Created also with BioRender.com.

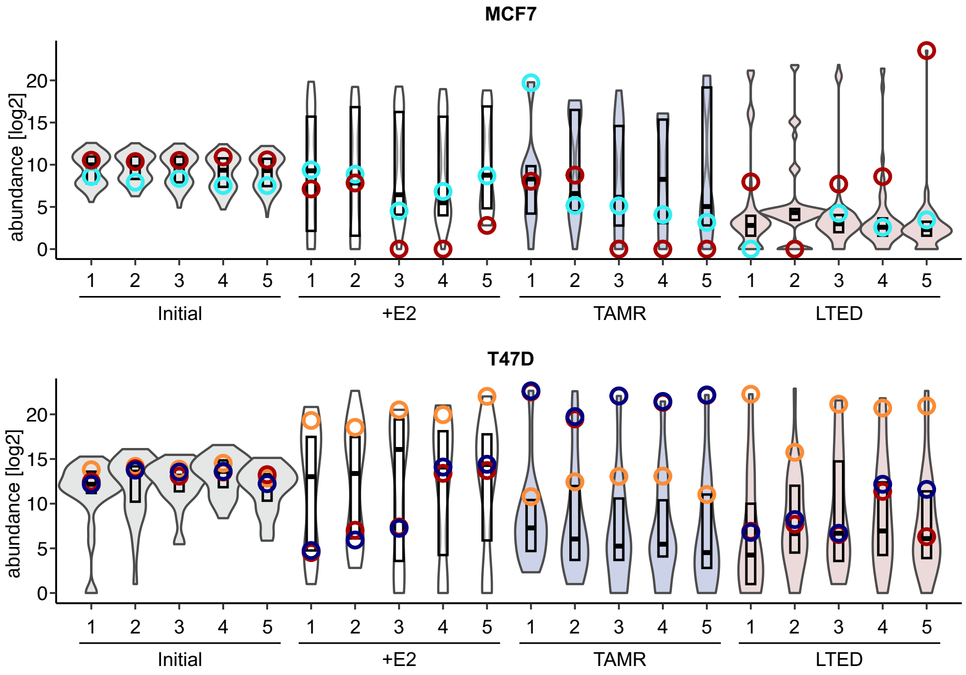
MCF7
abundance [log2]
0
5
10
15
20
1
2
3
4
5
Initial
+E2
TAMR
LTED
T47D
abundance [log2]
0
5
10
15
20
1
2
3
4
5
Initial
+E2
TAMR
LTED

Supplementary Figure 2: Barcode sequencing of replicates reveals an even barcode representation in the initial population, depletion of most, and enrichment of few barcodes after prolonged cultivation. Genomic DNA was isolated from barcoded MCF7 and T47D replicate cultures of the different conditions, i.e., initial cell pools (Initial), cells cultivated in media with estrogen (+E2), treated with 4-OHT (TAMR) or treated without estrogen (LTED). Then barcodes were PCR-amplified and sequenced. Violin plots of barcode reads whose sum were at least $5x10^5$ in all replicates are shown. Whisker plots represent the 25-75th percentile. For MCF7, one of the top five most enriched barcodes (*bright blue*) in the TAMR_1 replicate and the most enriched barcode for the LTED_5 replicate (*red*) are depicted. For T47D, the recurrently enriched *orange* (+E2 and LTED) and *dark blue* and *dark red* (TAMR replicates) barcodes are depicted. Color coding is according to Figure 1c.

a

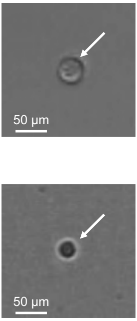

b

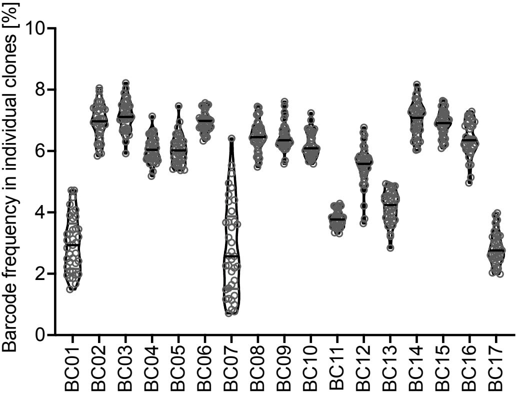

c

Integration locus	MCF7 LTED_5	T47D TAMR_2	T47D LTED_2
Chr4:110,457,931	505,127	–	–
Chr6:120,131,260	–	369,353	–
Chr3:189,684,206	–	21,445	–
Chr8:91,606,636	–	25,125	–
Chr7:110,418,502	–	–	176
Chr11:31,426,888	–	–	115

d

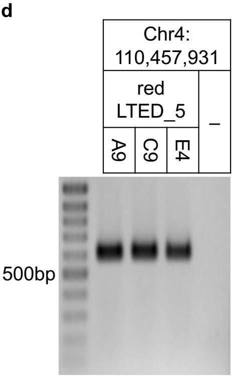

e

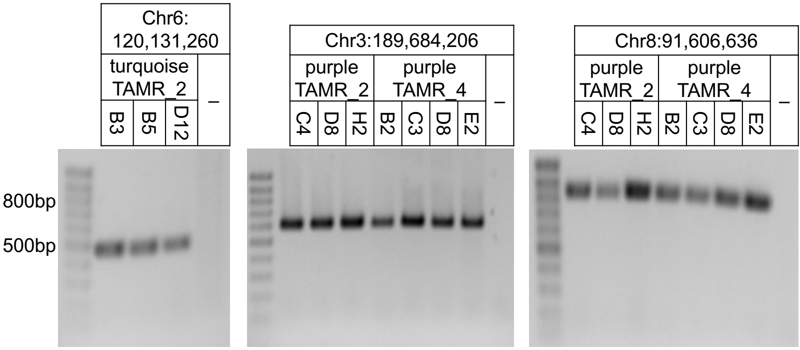

f

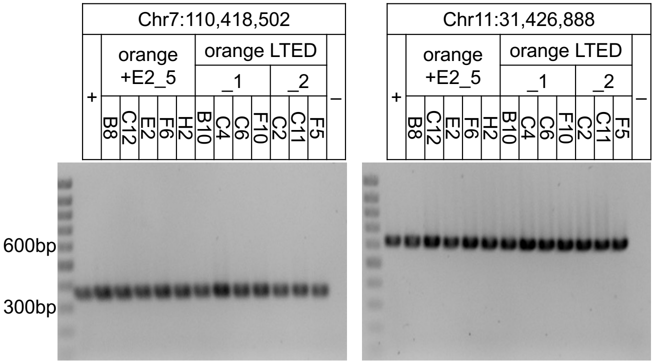

Supplementary Figure 3: Verification of barcode integration sites in MCF7 and T47D clones. Single cells were sorted from selected replicates, spotted into 96well plates, and cultivated until clones had grown out. **a.** Representative cell during spotting (top) and after having attached in a 96 well plate (bottom). **b.** Sequencing of 37 individual clones isolated from the MCF7 *blue* TAMR_1 replicate finds 17 integration events (BC01-BC17) as depicted in violin plots for each sequenced barcode. **c.** Barcode integration sites were identified for selected replicates with LAM-PCR [2] followed by sequencing. These integration sites were associated with specific barcodes based on read count data. '-' : Integration site not found. Verification of integration sites was done by amplification of barcodes using primers extending into the respective genomic loci in (**d.**) MCF7 *red* LTED_5 clones, (**e.**) T47D *light blue* and *purple* TAMR clones, and (**f**) T47D *orange* clones. **d.-f.**: +: Positive control (T47D LTED_2 pool). -: H_2O. A 100bp ladder was loaded left of the PCR amplification products in every gel.

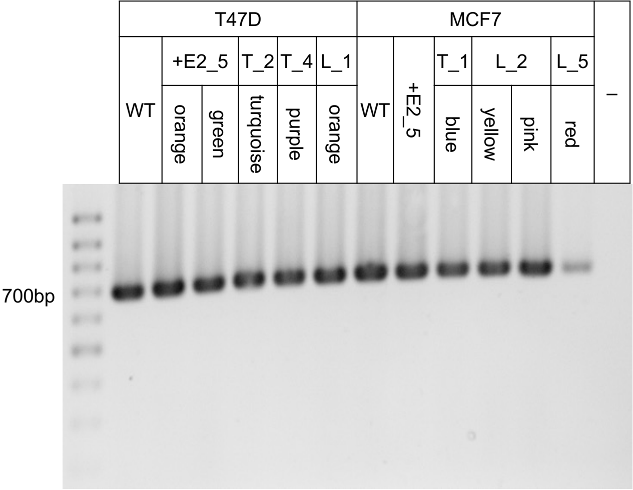

	NM_000125.4	CTCTATGAC
T47D	WT	CTCTATGAC
	orange +E2_5	CTCTATGAC
	green +E2_5	CTCTATGAC
	turquoise T_2	CTCTATGAC
	purple T_4	CTCTATGAC
	orange L_1	CTCTATGAC
MCF7	WT	CTCTATGAC
	+E2_5	CTCTATGAC
	blue T_1	CTCTATGAC
	yellow L_2	CTCTATGAC
	pink L_2	CTCTATGAC
	red L_5	CTCTATGAC

Supplementary Figure 4: Endocrine therapy resistance is not driven by *ESR1* hotspot mutations. Genomic sequence covering the *ESR1* hotspot codons 536-538 (Reference sequence: NM_000125.4) was PCR-amplified from control cell lines and single clones representing each of the different isolated populations (left panel), and then subjected to Sanger sequencing (right panel). Sequences were aligned using Snapgene with NM_000125.4. WT: respective non-barcoded T47D and MCF7 cell lines. T_2: TAMR_2. T_4: TAMR_4. L_1: LTED_1. T_1: TAMR_1. L_2: LTED_2. L_5: LTED_5. -: H_2O.

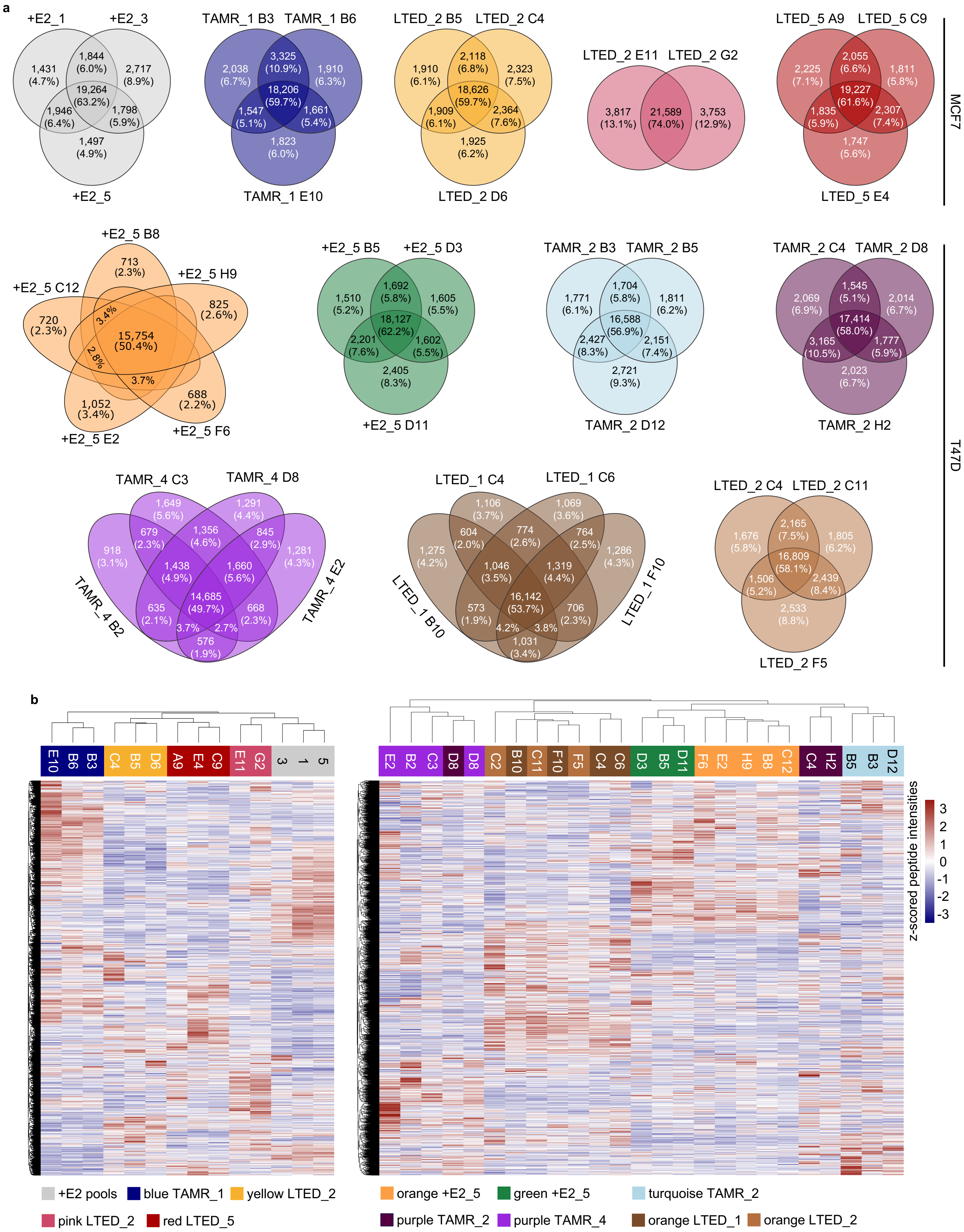

a
+E2_1
+E2_3
+E2_5
1,431 (4.7%)
1,844 (6.0%)
2,717 (8.9%)
19,264 (63.2%)
1,946 (6.4%)
1,798 (5.9%)
1,497 (4.9%)
TAMR_1 B3
TAMR_1 B6
TAMR_1 E10
2,038 (6.7%)
3,325 (10.9%)
1,910 (6.3%)
18,206 (59.7%)
1,547 (5.1%)
1,661 (5.4%)
1,823 (6.0%)
LTED_2 B5
LTED_2 C4
LTED_2 D6
1,910 (6.1%)
2,118 (6.8%)
2,323 (7.5%)
18,626 (59.7%)
1,909 (6.1%)
2,364 (7.6%)
1,925 (6.2%)
LTED_2 E11
LTED_2 G2
3,817 (13.1%)
21,589 (74.0%)
3,753 (12.9%)
LTED_5 A9
LTED_5 C9
LTED_5 E4
2,225 (7.1%)
2,055 (6.6%)
1,811 (5.8%)
19,227 (61.6%)
1,835 (5.9%)
2,307 (7.4%)
1,747 (5.6%)
MCF7
+E2_5 B8
+E2_5 H9
+E2_5 C12
+E2_5 E2
+E2_5 F6
713 (2.3%)
825 (2.6%)
720 (2.3%)
3.4%
15,754 (50.4%)
2.8%
3.7%
688 (2.2%)
1,052 (3.4%)
+E2_5 B5
+E2_5 D3
+E2_5 D11
1,510 (5.2%)
1,692 (5.8%)
1,605 (5.5%)
18,127 (62.2%)
2,201 (7.6%)
1,602 (5.5%)
2,405 (8.3%)
TAMR_2 B3
TAMR_2 B5
TAMR_2 D12
1,771 (6.1%)
1,704 (5.8%)
1,811 (6.2%)
16,588 (56.9%)
2,427 (8.3%)
2,151 (7.4%)
2,721 (9.3%)
TAMR_2 C4
TAMR_2 D8
TAMR_2 H2
2,069 (6.9%)
1,545 (5.1%)
2,014 (6.7%)
17,414 (58.0%)
3,165 (10.5%)
1,777 (5.9%)
2,023 (6.7%)
TAMR_4 C3
TAMR_4 D8
TAMR_4 B2
TAMR_4 E2
1,649 (5.6%)
1,291 (4.4%)
679 (2.3%)
1,356 (4.6%)
845 (2.9%)
918 (3.1%)
1,281 (4.3%)
1,438 (4.9%)
1,660 (5.6%)
14,685 (49.7%)
635 (2.1%)
668 (2.3%)
3.7%
2.7%
576 (1.9%)
LTED_1 C4
LTED_1 C6
LTED_1 B10
LTED_1 F10
1,106 (3.7%)
1,069 (3.6%)
604 (2.0%)
774 (2.6%)
764 (2.5%)
1,275 (4.2%)
1,286 (4.3%)
1,046 (3.5%)
1,319 (4.4%)
16,142 (53.7%)
573 (1.9%)
706 (2.3%)
4.2%
3.8%
1,031 (3.4%)
LTED_2 C4
LTED_2 C11
LTED_2 F5
1,676 (5.8%)
2,165 (7.5%)
1,805 (6.2%)
16,809 (58.1%)
1,506 (5.2%)
2,439 (8.4%)
2,533 (8.8%)
T47D
b
E10 B6 B3 C4 B5 D6 A9 E4 C9 E11 G2 3 1 5
E2 B2 C3 D8 D8 C2 B10 C11 F10 F5 C4 C6 D3 B5 D11 F6 E2 H9 B8 C12 C4 H2 B5 B3 D12
z-scored peptide intensities
3
2
1
0
-1
-2
-3
+E2 pools
blue TAMR_1
yellow LTED_2
pink LTED_2
red LTED_5
orange +E2_5
green +E2_5
turquoise TAMR_2
purple TAMR_2
purple TAMR_4
orange LTED_1
orange LTED_2

Supplementary Figure 5: Phosphoprotein profiles are stable only in clones carrying the same barcodes. a. Unbiased MS-based phosphoproteomic analysis was performed with the indicated bulk populations (+E2 for MCF7), and individual clones (+E2 for T47D, and respective endocrine therapy-resistant TAMR and LTED clones for MCF7 as well as T47D). Venn diagrams show the numbers and percentages of identified phosphosites in the indicated biological replicates. For T47D *orange* +E2_5 clones, only overlap larger than 2.0% are indicated and only selected number of phosphosites are indicated for better visualization. **b.** Heatmaps presenting consistently identified phosphosites in rows and cell lines in columns were generated using the R package Pheatmap (version 1.0.12) for MCF7 (left panel) and T47D (right panel). Hierarchical clustering was applied in both phosphosites (rows) and cell lines (columns) (Euclidean distance). Shown are z-scored peptide intensities.

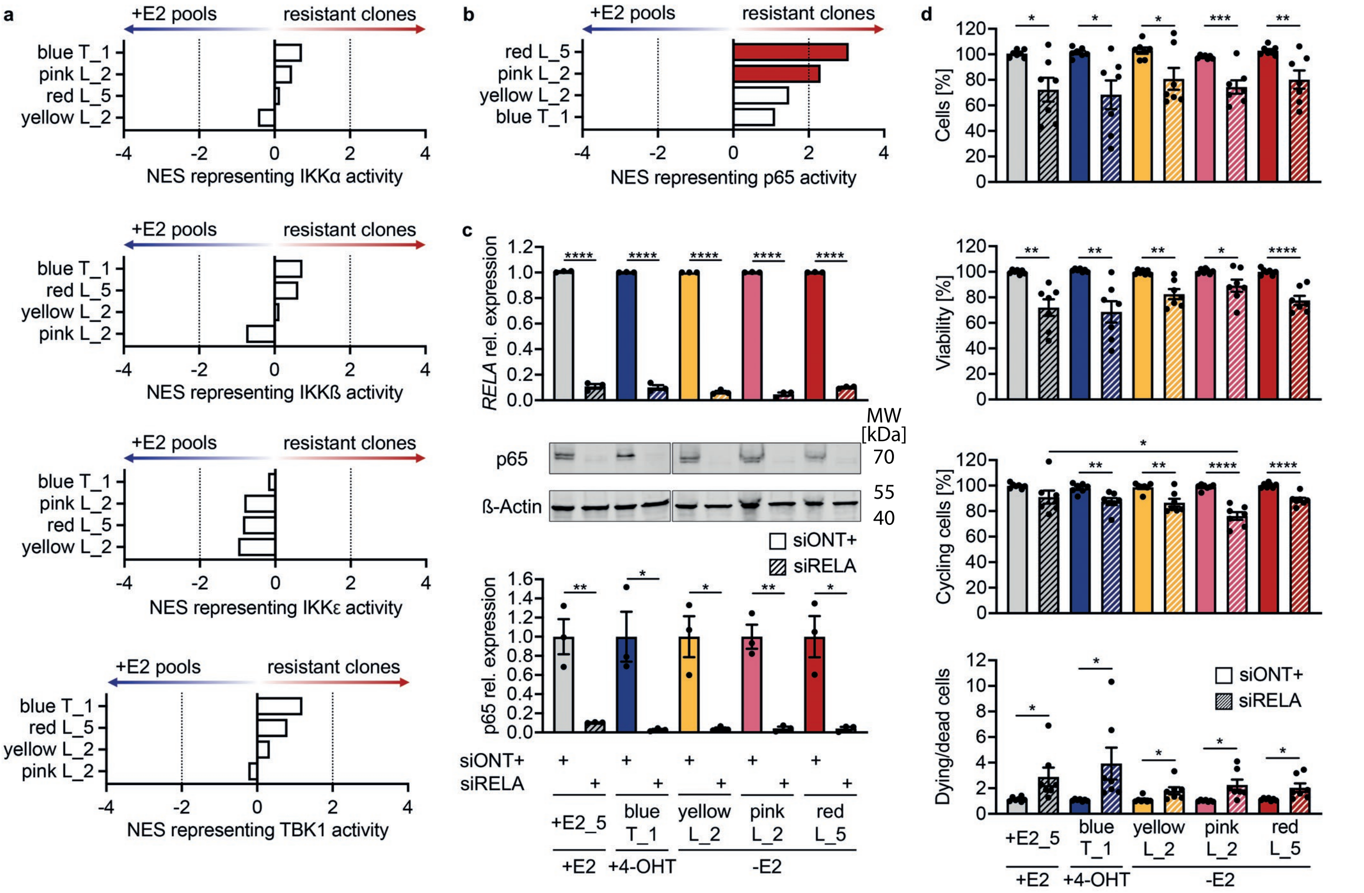

a
+E2 pools
resistant clones
blue T_1
pink L_2
red L_5
yellow L_2
NES representing IKKα activity
NES representing IKKß activity
NES representing IKKε activity
NES representing TBK1 activity
b
NES representing p65 activity
c
RELA rel. expression
MW [kDa]
p65
ß-Actin
70
55
40
siONT+
siRELA
p65 rel. expression
+E2_5
blue T_1
yellow L_2
pink L_2
red L_5
+E2
+4-OHT
-E2
d
Cells [%]
Viability [%]
Cycling cells [%]
Dying/dead cells

Supplementary Figure 6: Knockdown of *RELA*/p65 affects endocrine therapy resistant, however, also sensitive cells. **a.** Phosphoproteomic data of MCF7 +E2 cell pools and resistant clones was analyzed [3,4] to infer relative activities of indicated kinases capable of phosphorylating IκBα, in respective resistant clones compared to control +E2 cell pools. **b.** Analysis of p65 activity from RNA-seq data [4,5]. **c.** Validation of knockdown efficiency of *RELA* mRNA (upper panel) and p65 protein (lower panel) 6 days post transfection of *RELA* siRNA or non-targeting control (siONT+). Upper panel: *RELA* expression was normalized to the expression of the housekeeping genes *ACTB* and *GAPDH*. Relative expression was then normalized to the non-targeting controls (siONT+). n = 3 with 3 technical replicates each. Lower panel: Representative Western Blot and quantification of three biological replicates. p65 protein intensities were normalized to ß-Actin protein intensities. Shown are mean ± SEM. * p<0.05, ** p<0.01, **** p<0.0001 as determined by unpaired two-tailed t-test. MCF7 +E2_5 (□), TAMR_1 B6 (*blue* T_1, ■), LTED_2 C4 (*yellow* L_2, ■), LTED_2 E11 (*pink* L_2, ■) and LTED_5 C9 (*red* L_5, ■). **d** Phenotypic analysis of *RELA*/p65 effects. Indicated cell clones were transfected with siRELA or control (siONT+). Nuclear cell count and ATP levels were determined 6 days after transfection, while EdU and DAPI incorporation were assessed 4 days after transfection (n = 7 with ≥ 4 technical replicates each). Data was normalized to non-targeting control siRNA (siONT+). Shown are mean ± SEM. * p<0.05, ** p<0.01, *** p<0.001, **** p<0.0001 as determined by two-tailed t-tests (siONT+ vs. siRELA) or one-way ANOVA with Dunnett multiple comparisons test (siRELA transfected conditions).

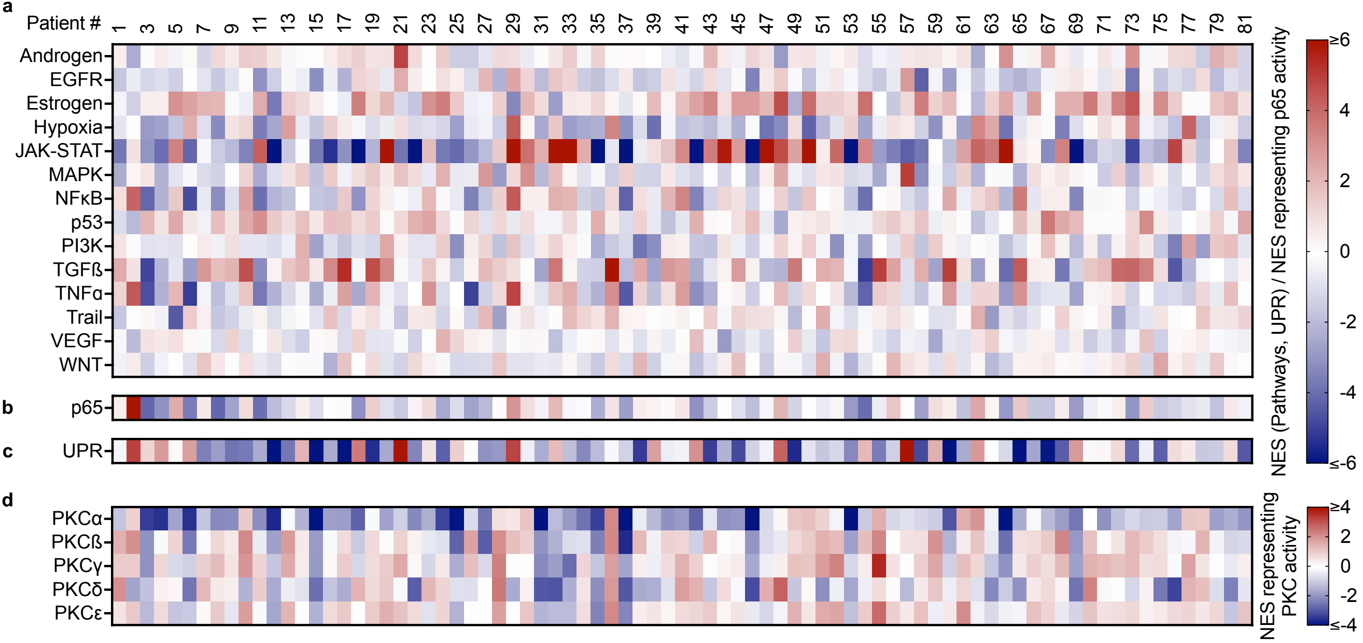

a
Patient #
1
3
5
7
9
11
13
15
17
19
21
23
25
27
29
31
33
35
37
39
41
43
45
47
49
51
53
55
57
59
61
63
65
67
69
71
73
75
77
79
81
Androgen
EGFR
Estrogen
Hypoxia
JAK-STAT
MAPK
NFκB
p53
PI3K
TGFß
TNFα
Trail
VEGF
WNT
b
p65
c
UPR
d
PKCα
PKCß
PKCγ
PKCδ
PKCε
NES (Pathways, UPR) / NES representing p65 activity
≥6
4
2
0
-2
-4
≤-6
NES representing PKC activity
≥4
2
0
-2
≤-4

Supplementary Figure 7: Activities in patients with ER+ disease. Patients with ER+ (n=81) disease were selected in the CPTAC-BRCA cohort and indicated activities were calculated on a per patient basis. For pathway (**a.**), p65 (**b.**) and UPR (**c**) activation, CPTAC RNA-seq data were z-scored before further analyses by PROGENy [6,7] (**a.**), DoRothEA [5] and decoupleR [4] (**b.)** and ssGSEA [8] (**c.**). PKC activity estimates (**d.**) were based on CPTAC phosphoproteomic data [3,4,9]. **a.**-**d.** Significance is not indicated to improve visualization.

Supplementary Tables

Supplementary Table 1: Numbers of barcode reads. Barcodes were sequenced in bulk populations of five replicates each of MCF7 and T47D cells at the initial time point, and after eight months of cultivation in media containing estrogen (+E2), 4-OH-tamoxifen (TAMR), or that was deprived of estrogen (LTED). Counts of barcode reads are given in million ($x10^6$).

Cell line		MCF7				T47D			
Treatment		Initial	+E2	TAMR	LTED	Initial	+E2	TAMR	LTED
Replicate	1	15.71	14.60	14.43	13.18	12.27	8.42	13.08	9.98
	2	15.61	14.61	20.75	15.59	18.50	12.67	11.67	8.52
	3	15.06	14.79	13.83	11.56	13.90	11.77	9.77	9.43
	4	12.80	11.05	16.78	13.30	23.35	10.56	5.90	8.72
	5	12.66	12.40	23.92	12.57	13.07	8.36	9.74	9.14

Supplementary Table 2: Isolated clones and barcode sequences. Individual cells were isolated from selected replicates and transferred into 96well plates (one plate/replicate). Barcodes were sequenced from either using Sanger or NGS technologies, depending on the number of barcodes in a given clone. The barcode frequency refers to the representation of a given barcode in the bulk-sequencing data of the respective replicate. Cultivation of selected clones was continued, when several clones carrying the same barcode(s) had been identified. These clones and barcodes received a virtual color code.

Barcode sequence	Replicate	Barcode frequency	Color coding	Individual clones
ACAGAGTGAGAGTGTGAGTCTGAGTCACAG	MCF7 TAMR_1	5.10%	*blue*	B3, B6, E10
ACTGTGTGTGACAGAGACAGAACTCTGACA		Not detected		
AGAGACTCAGTCTGTGAGACTGACACAGTC		5.46%		
AGTCTGTCTGTGTCTCACAGAGTGTGAGAC		5.43%		
AGTCTGTGTGTCTCTGTCTGTCTCTGAGTG		4.78%		
AGTGAGAGTGTCAGTCTGACTGACACACAG		5.77%		
TCAGTGAGAGTGAGTCTCAGTCTGAGTGTG		6.23%		
TCTGTGTGTGAGTGACTGAGTGTCAGTGTG		5.94%		
TGACACTGAGAGAGAGAGTGACACAGTGAC		5.77%		
TGACAGAGTGTGTCTGAGAGTGAGTGAGTG		5.85%		
TGACTGTGTGTGCAGAGAGTGAGTGTGTCA		Not detected		
TGTCAGAGTGAGTGTGACTCACTCAGAGTC		6.10%		
TGTCTGTCAGACTGTGAGAGAGAGTCAGAC		3.80%		
TGTCTGTGTGACAGTCACACTGTCTCAGTC		6.29%		
TGTGAGTGTCACTGACTGTCTGAGAGTGAG		6.21%		
TGTGTGAGACTGTCTCACAGTCAGAGAGAC		6.01%		
TGTGTGTCAGAGTGTCTGACAGAGAGTGAG		4.96%		
AGACACAGAGTGAGAGAGAGACACAGAGTG	MCF7 LTED_2	21.17%	*yellow*	B5, C4, D6
AGAGTGTGTCTGTGTGTGACTGACAGTCTG		23.81%		
AGTCTGTGAGTGTGTGTCTCACTGACTGTG		22.77%		
TCTGTCACACTGCGACACTCTCAGTCAGTC		Not detected		
TGTGTCACACACTGACTCTGTCAGTGAGTG		19.82%		
ACAGTGTGTCTGAGACACAGTGACACAGTG	MCF7 LTED_2	Not detected	*pink*	E11, G2
AGTCTCAGAGTGAGTCTGAGTGTCTGTGTC		Not detected		
AGTGTCTCAGTGTCTGTCAGTGACACTCTG		Not detected		
TGACTGAGAGTGAGACACACTGTCACACAC		Not detected		
TGACTGAGTCTGAGTCTGAGAGAGTGTCTC		Not detected		
TGTCTCTCAGAGTGAGTGAGAGTGACTCAC		Not detected		
AGACTCTGAGACTGTCTGTGAGTGACTCAC	MCF7 LTED_5	96.55%	*red*	A9, C9, E4
AGACTCTCTGTGACAGAGACTCTGTGTGAG	T47D +E2_5 and LTED	50.46% in +E2_5	*orange*	+E2_5: B8, C12, E2, F6, H9<br>LTED_1: B10, C4, C6, F10<br>LTED_2: C2, C11, F5
AGACTCTGTGTCACACTGAGTCTGAGTGTG	T47D +E2_5	3.44%	*green*	B5, D3, D11
TGTGACTGTGAGACACAGAGAGTGAGTGTG	T47D TAMR_2	26.92%	*turquoise*	B3, B5, D12
AGAGACAGTGTGACTGTCACTGAGAGAGAG	T47D TAMR_2 and TAMR_4	44.98% in TAMR_4	*dark red/purple*	TAMR_2: C4, D8, H2<br>TAMR_4: B2, C3, D8, E2
ACTGAGACTGAGTCACACTGTCACAGTGAC	T47D TAMR_2 and TAMR_4	48.52% in TAMR_4	*dark blue/purple*	TAMR_2: C4, D8, H2<br>TAMR_4: B2, C3, D8, E2

Supplementary Table 3: Association of integration sites with individual clones. LAM-PCR [2] followed by sequencing was performed with five selected replicates (MCF7: TAMR_2, LTED_5; T47D: +E2_2, TAMR_2, LTED_2) to identify chromosomal positions where virus had integrated. Color codes of barcodes are indicated when these could be associated with particular viral integration sites. Integration sites that could not be associated with any barcode are indicated with n.d. 'Primer sequence (5'-3')' denotes sequences at the genomic locus where the viral DNA had integrated and that were used to amplify integration sites in particular clones (compare Supplementary Figure 3). The universal primer 5'-CCCAACGAAGATAAGATCTGC-3' maps to the viral LTR and was used as second primer in all PCR.

Integration locus	Replicate	Color code of barcode	Primer sequence (5'-3')
Chr3:17,402,189	T47D +E2_2 and LTED_2	n.d.	CGACTGAGTCATAGGGTGAGG
Chr3:189,684,206	T47D TAMR_2	*purple*	TCCCTTGACATCACTAATAGGC
Chr4:110,457,931	MCF7 LTED_5	*red*	GTTACCTCTGCTTGGCGAAC
Chr4:112,000,728	T47D +E2_2 and LTED_2	n.d.	ACTCACATTGAGGCTAAAGGG
Chr6:120,131,260	T47D TAMR_2	*turquise*	CTTGCTGAAATTAAGGAGAGACG
Chr7:110,418,502	T47D +E2_2 and LTED_2	*orange*	ACCAAAATCCTCTCGGGCTG
Chr8:66,103,520	T47D TAMR_2	n.d.	CTGAGGCCACAATATTAACAGC
Chr8:91,606,636	T47D TAMR_2	*purple*	GAGATTCAGCAGCAATAATCAGG
Chr11:31,426,888	T47D +E2_2 and LTED_2	*orange*	GCTTGTGACCCACGTCTTAG

Supplementary Figure 8: Uncropped blots showing data presented in Figure 6b

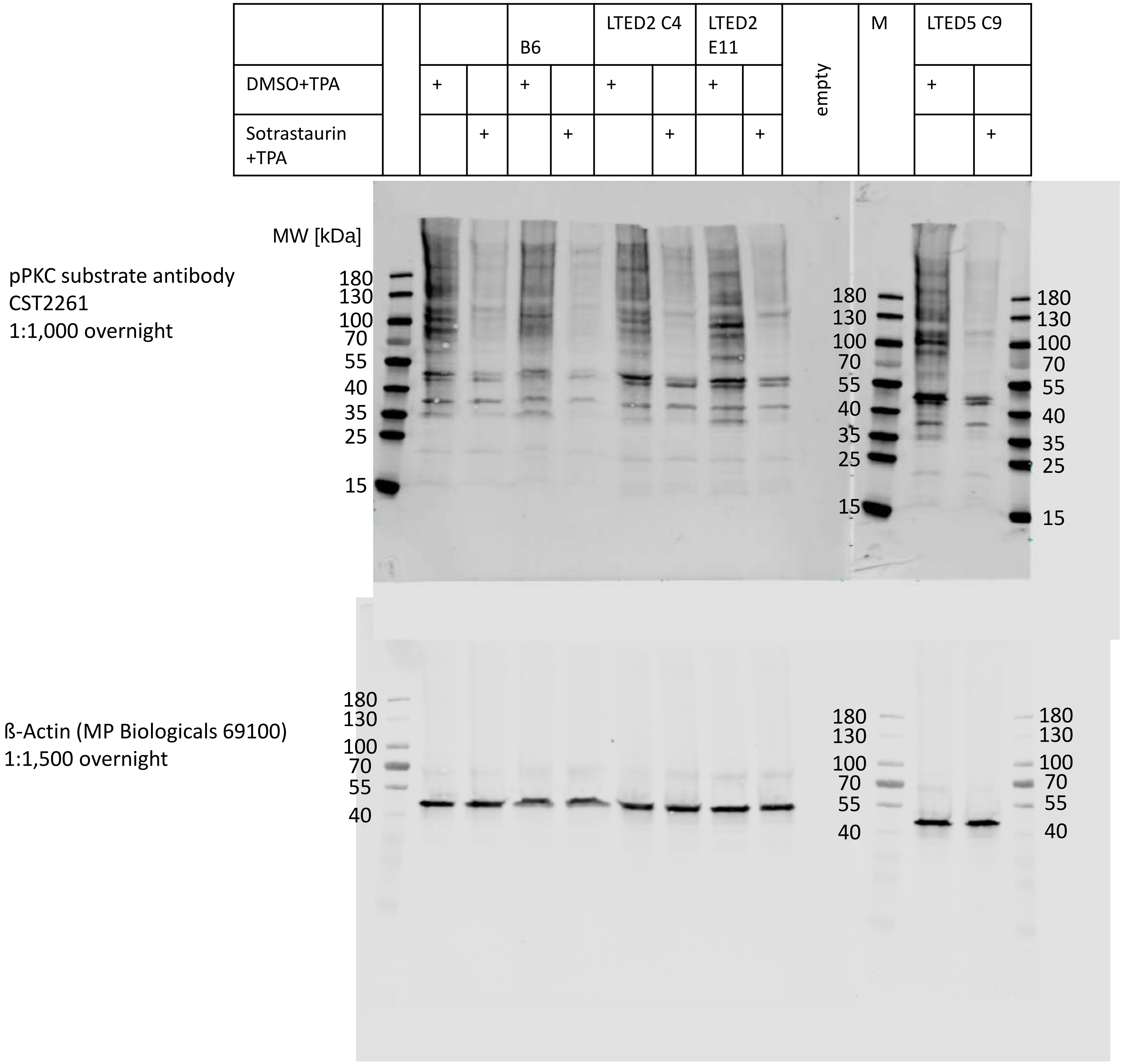

Marker: PageRuler Prestained Protein ladder (Thermo Fisher Scientific, REF: 26616)

Comments:

- the 70kDa band of the marker is only faintly visible in the 680 channel (because it's red)
- The ACTB signal is very strong so some parts of the marker may be a little faint

Legend: E2_5: +E2_5; T1B6: TAMR_1 B6; L2C4: LTED_2 C4; L2E11: LTED_2 E11; L5C9: LTED_5 C9

Supplementary Figure 9: Uncropped blots showing data presented in Supplementary Figure 6c

Cell line	M	E2_5		T1B6		M	L2C4		L2E11		L5C9		empty	empty	M
siONT		+		+			+		+		+				
siRELA			+		+			+		+		+			

NFkB (CST8242, 1:1,000)

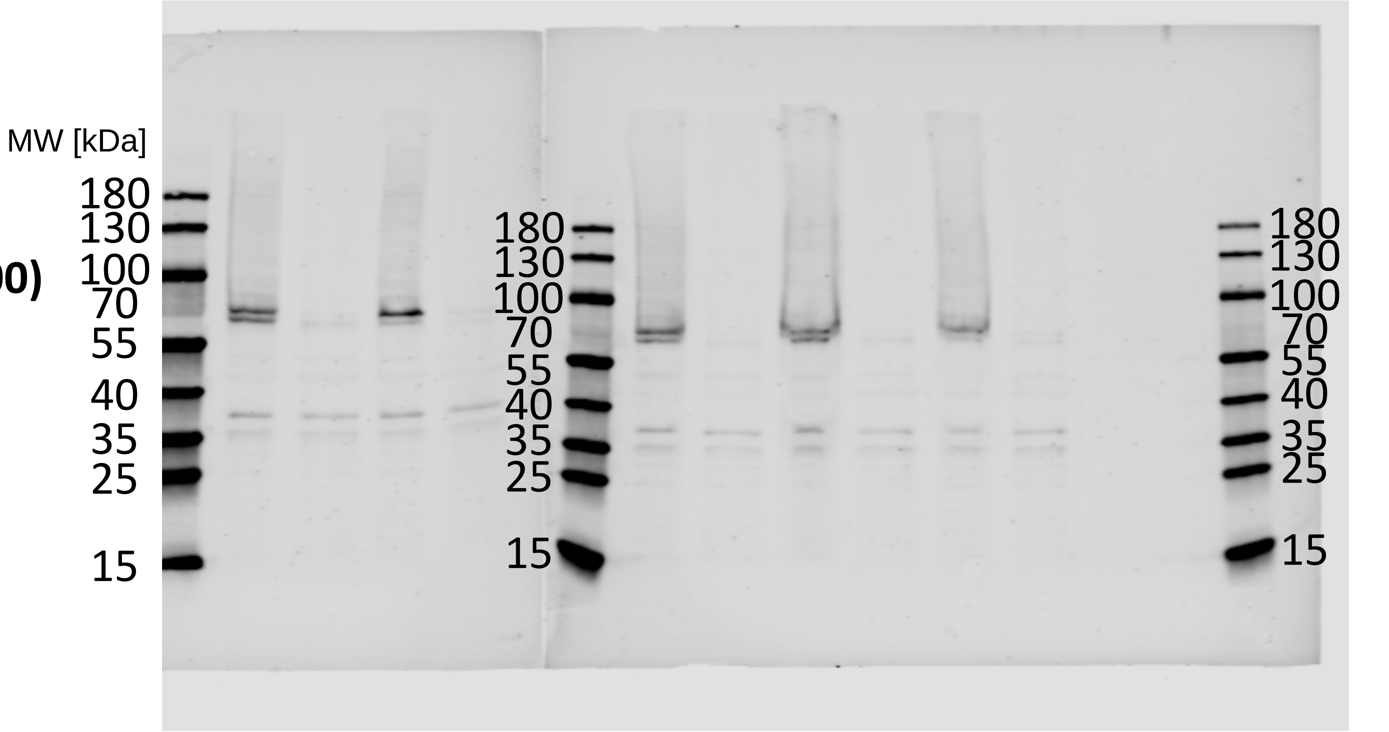

ACTB (MP691000, 1:1,2500)

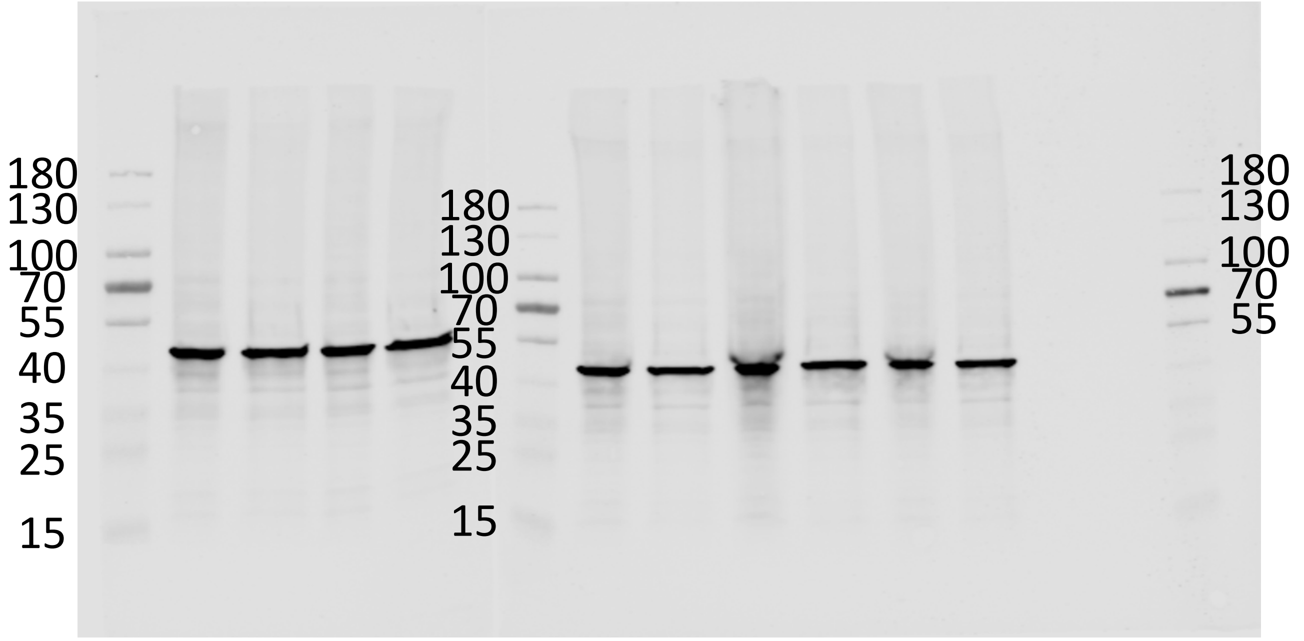

Supplementary References

1 Bhang, H. E. *et al.* Studying clonal dynamics in response to cancer therapy using high-complexity barcoding. *Nat Med* **21**, 440-448, doi:10.1038/nm.3841 (2015).

2 Schmidt, M. *et al.* Detection and direct genomic sequencing of multiple rare unknown flanking DNA in highly complex samples. *Human gene therapy* **12**, 743-749, doi:10.1089/104303401750148649 (2001).

3 Turei, D. *et al.* Integrated intra- and intercellular signaling knowledge for multicellular omics analysis. *Mol Syst Biol* **17**, e9923, doi:10.15252/msb.20209923 (2021).

4 Badia-i-Mompel, P. *et al.* decoupleR: ensemble of computational methods to infer biological activities from omics data. *Bioinform Adv* **2**, vbac016, doi:10.1093/bioadv/vbac016 (2022).

5 Garcia-Alonso, L., Holland, C. H., Ibrahim, M. M., Turei, D. & Saez-Rodriguez, J. Benchmark and integration of resources for the estimation of human transcription factor activities. *Genome Res.* **29**, 1363-1375, doi:10.1101/gr.240663.118 (2019).

6 Schubert, M. *et al.* Perturbation-response genes reveal signaling footprints in cancer gene expression. *Nat Commun* **9**, 20, doi:10.1038/s41467-017-02391-6 (2018).

7 Holland, C. H., Szalai, B. & Saez-Rodriguez, J. Transfer of regulatory knowledge from human to mouse for functional genomics analysis. *Biochim Biophys Acta Gene Regul Mech* **1863**, 194431, doi:10.1016/j.bbagrm.2019.194431 (2020).

8 Barbie, D. A. *et al.* Systematic RNA interference reveals that oncogenic KRAS-driven cancers require TBK1. *Nature* **462**, 108-112, doi:10.1038/nature08460 (2009).

9 Krug, K. *et al.* Proteogenomic Landscape of Breast Cancer Tumorigenesis and Targeted Therapy. *Cell* **183**, 1436-1456 e1431, doi:10.1016/j.cell.2020.10.036 (2020).
