# Supplementary material for: Clonal heterogeneity in ER+ breast cancer reveals the proteasome and PKC as potential therapeutic targets
Source: NPJ Breast Cancer. 2023 Dec 2;9:97. doi: 10.1038/s41523-023-00604-4 (PMC10693625; doi:10.1038/s41523-023-00604-4)
Supplement: Supplementary file 2 — nr-reporting-summary [file 41523_2023_604_MOESM2_ESM.pdf]

nature portfolio

Corresponding author(s): Stefan Wiemann, Lukas Beumers

Last updated by author(s): Jul 5, 2023

Reporting Summary

Nature Portfolio wishes to improve the reproducibility of the work that we publish. This form provides structure for consistency and transparency in reporting. For further information on Nature Portfolio policies, see our Editorial Policies and the Editorial Policy Checklist.

Statistics

For all statistical analyses, confirm that the following items are present in the figure legend, table legend, main text, or Methods section.

n/a	Confirmed	
☐	☒	The exact sample size (*n*) for each experimental group/condition, given as a discrete number and unit of measurement
☐	☒	A statement on whether measurements were taken from distinct samples or whether the same sample was measured repeatedly
☐	☒	The statistical test(s) used AND whether they are one- or two-sided<br>*Only common tests should be described solely by name; describe more complex techniques in the Methods section.*
☒	☐	A description of all covariates tested
☐	☒	A description of any assumptions or corrections, such as tests of normality and adjustment for multiple comparisons
☐	☒	A full description of the statistical parameters including central tendency (e.g. means) or other basic estimates (e.g. regression coefficient) AND variation (e.g. standard deviation) or associated estimates of uncertainty (e.g. confidence intervals)
☐	☒	For null hypothesis testing, the test statistic (e.g. *F*, *t*, *r*) with confidence intervals, effect sizes, degrees of freedom and *P* value noted<br>*Give P values as exact values whenever suitable.*
☒	☐	For Bayesian analysis, information on the choice of priors and Markov chain Monte Carlo settings
☐	☒	For hierarchical and complex designs, identification of the appropriate level for tests and full reporting of outcomes
☒	☐	Estimates of effect sizes (e.g. Cohen's *d*, Pearson's *r*), indicating how they were calculated

Our web collection on statistics for biologists contains articles on many of the points above.

Software and code

Policy information about availability of computer code

Data collection	The software and versions used to capture data are described in detail in the Methods
Data analysis	The software and versions used to analyze data are described in detail in the Methods. A link to GitHub is provided where own code has been deposited

For manuscripts utilizing custom algorithms or software that are central to the research but not yet described in published literature, software must be made available to editors and reviewers. We strongly encourage code deposition in a community repository (e.g. GitHub). See the Nature Portfolio guidelines for submitting code & software for further information.

Data

Policy information about availability of data

All manuscripts must include a data availability statement. This statement should provide the following information, where applicable:

- Accession codes, unique identifiers, or web links for publicly available datasets
- A description of any restrictions on data availability
- For clinical datasets or third party data, please ensure that the statement adheres to our policy

RNA-seq data from cell lines has been deposited in the EGA (at EBI). Access to this (human) sequencing data is controlled (DAC). All non-sensitive data has been deposited in appropriate databases (PRIDE for MS-data, Zenodo for other datatypes). That data is freely accessible.

Research involving human participants, their data, or biological material

Policy information about studies with human participants or human data. See also policy information about sex, gender (identity/presentation), and sexual orientation and race, ethnicity and racism.

Reporting on sex and gender	We only considered breast cancer in females
Reporting on race, ethnicity, or other socially relevant groupings	does not apply
Population characteristics	does not apply
Recruitment	does not apply
Ethics oversight	does not apply

Note that full information on the approval of the study protocol must also be provided in the manuscript.

Field-specific reporting

Please select the one below that is the best fit for your research. If you are not sure, read the appropriate sections before making your selection.

☒ Life sciences ☐ Behavioural & social sciences ☐ Ecological, evolutionary & environmental sciences

For a reference copy of the document with all sections, see nature.com/documents/nr-reporting-summary-flat.pdf

Life sciences study design

All studies must disclose on these points even when the disclosure is negative.

Sample size	does not apply
Data exclusions	no data was excluded from analysis
Replication	at least three biological replicates were analyzed for every condition. At least three technical replicates was analyzed for every biological replicate
Randomization	no randomization was done as we deemed this not necessary
Blinding	no blinding was done as we deemed this not necessary (or even a potential source of error)

Reporting for specific materials, systems and methods

We require information from authors about some types of materials, experimental systems and methods used in many studies. Here, indicate whether each material, system or method listed is relevant to your study. If you are not sure if a list item applies to your research, read the appropriate section before selecting a response.

Materials & experimental systems

n/a	Involved in the study	
☐	☒	Antibodies
☐	☒	Eukaryotic cell lines
☐	☐	Palaeontology and archaeology
☐	☐	Animals and other organisms
☐	☒	Clinical data
☐	☐	Dual use research of concern
☐	☐	Plants

Methods

n/a	Involved in the study	
☐	☐	ChIP-seq
☐	☐	Flow cytometry
☐	☐	MRI-based neuroimaging

Antibodies

Antibodies used	primary antibodies: anti beta-actin (0869100-CF, MP Biologicals, Irvine, CA, USA); anti p65 (CST8242, Cell Signaling Technology, Danvers, MA, USA); p(Ser) PKC Substrate (CST2261 Cell Signaling Technology); secondary antibodies: anti-mouse (SA5-35521, Thermo Fisher Scientific), anti-rabbit antibodies (A-21077, Thermo Fisher Scientific)

Validation	the antibodies were validated in Western blots with breast cancer cell lines (HCC1954, MDA-MB-231, SKBR3, MCF7), either serum-starved or harvested 15 minutes after stimulation with EGF or HRG. Antibodies detecting single bands at the expected size or no bands at all were rated "good" and "acceptable", respectively. Lack of detection of a particular protein/phosphosite comes with no/low background signal. Detection of that band in some other condition suggests specificity of detection, provided the band is single and at the expected apparent MW.

Eukaryotic cell lines

Policy information about cell lines and Sex and Gender in Research

Cell line source(s)	MCF7 (Cellosaurus CVCL_0031) and T47D (CVCL_0553) were obtained from ATCC (LGC Standards GmbH, Wesel, Germany) and had been derived from female breast cancer patients. HEK293FT (Cellosaurus CVCL_6911) was obtained from Thermo Fisher Scientific (Waltham, MA, USA).
Authentication	STR profiling (Multiplexion GmbH Heidelberg, Germany)
Mycoplasma contamination	The cell lines were tested on a regular basis (once every month) throughout the study, using a PCR-based assay (has positive and negative controls). The cell lines were tested negative at all times during the study.
Commonly misidentified lines (See ICLAC register)	does not apply

Palaeontology and Archaeology

Specimen provenance	does not apply
Specimen deposition	does not apply
Dating methods	does not apply

☐ Tick this box to confirm that the raw and calibrated dates are available in the paper or in Supplementary Information.

Ethics oversight	does not apply

Note that full information on the approval of the study protocol must also be provided in the manuscript.

Animals and other research organisms

Policy information about studies involving animals; ARRIVE guidelines recommended for reporting animal research, and Sex and Gender in Research

Laboratory animals	does not apply
Wild animals	does not apply
Reporting on sex	does not apply
Field-collected samples	does not apply
Ethics oversight	does not apply

Note that full information on the approval of the study protocol must also be provided in the manuscript.

Clinical data

Policy information about clinical studies
All manuscripts should comply with the ICMJE guidelines for publication of clinical research and a completed CONSORT checklist must be included with all submissions.

Clinical trial registration	does not apply
Study protocol	does not apply
Data collection	does not apply
Outcomes	does not apply

Dual use research of concern

Policy information about dual use research of concern

Hazards

Could the accidental, deliberate or reckless misuse of agents or technologies generated in the work, or the application of information presented in the manuscript, pose a threat to:

No	Yes	
☒	☐	Public health
☒	☐	National security
☒	☐	Crops and/or livestock
☒	☐	Ecosystems
☒	☐	Any other significant area

Experiments of concern

Does the work involve any of these experiments of concern:

No	Yes	
☒	☐	Demonstrate how to render a vaccine ineffective
☒	☐	Confer resistance to therapeutically useful antibiotics or antiviral agents
☒	☐	Enhance the virulence of a pathogen or render a nonpathogen virulent
☒	☐	Increase transmissibility of a pathogen
☒	☐	Alter the host range of a pathogen
☒	☐	Enable evasion of diagnostic/detection modalities
☒	☐	Enable the weaponization of a biological agent or toxin
☒	☐	Any other potentially harmful combination of experiments and agents

Plants

Seed stocks	does not apply
Novel plant genotypes	does not apply
Authentication	does not apply

ChIP-seq

Data deposition

☐ Confirm that both raw and final processed data have been deposited in a public database such as GEO.

☐ Confirm that you have deposited or provided access to graph files (e.g. BED files) for the called peaks.

Data access links<br>*May remain private before publication.*	does not apply
Files in database submission	does not apply
Genome browser session<br>(e.g. UCSC)	does not apply

Methodology

Replicates	does not apply
Sequencing depth	does not apply
Antibodies	does not apply

Peak calling parameters	does not apply
Data quality	does not apply
Software	does not apply

Flow Cytometry

Plots

Confirm that:

- [ ] The axis labels state the marker and fluorochrome used (e.g. CD4-FITC).
- [ ] The axis scales are clearly visible. Include numbers along axes only for bottom left plot of group (a 'group' is an analysis of identical markers).
- [ ] All plots are contour plots with outliers or pseudocolor plots.
- [ ] A numerical value for number of cells or percentage (with statistics) is provided.

Methodology

Sample preparation	does not apply
Instrument	does not apply
Software	does not apply
Cell population abundance	does not apply
Gating strategy	does not apply

- [ ] Tick this box to confirm that a figure exemplifying the gating strategy is provided in the Supplementary Information.

Magnetic resonance imaging

Experimental design

Design type	does not apply
Design specifications	does not apply
Behavioral performance measures	does not apply

Acquisition

Imaging type(s)	does not apply
Field strength	does not apply
Sequence & imaging parameters	does not apply
Area of acquisition	does not apply

Diffusion MRI ☐ Used ☒ Not used

Preprocessing

Preprocessing software	does not apply
Normalization	does not apply
Normalization template	does not apply
Noise and artifact removal	does not apply
Volume censoring	does not apply

Statistical modeling & inference

Model type and settings	does not apply
Effect(s) tested	does not apply

Specify type of analysis: ☐ Whole brain ☐ ROI-based ☐ Both

Statistic type for inference (See Eklund et al. 2016)	does not apply
Correction	does not apply

Models & analysis

n/a	Involved in the study
☒	☐ Functional and/or effective connectivity
☒	☐ Graph analysis
☒	☐ Multivariate modeling or predictive analysis
